# Drug Repositioning in Kinase Inhibitor Discovery for Cancer Therapy

**DOI:** 10.3390/pharmaceutics18101240

**Published:** 2026-09-29

**Authors:** Qianran Sun, Xiaocong Pang, Wenwu Liu

**Affiliations:** 1 Department of Pharmacy, Peking University First Hospital, Beijing 100034, China; sunqianran@bjmu.edu.cn; 2 State Key Laboratory of Natural and Biomimetic Drugs, School of Pharmaceutical Sciences, Peking University, Beijing 100191, China

**Keywords:** drug repurposing, kinase inhibitor, oncology, artificial intelligence

## Abstract

Repurposing of existing drugs has emerged as a time- and cost-effective strategy in oncology drug discovery. Given the critical role of dysregulated kinase signaling in cancer, kinase-focused repurposing strategies have attracted considerable interest. This approach encompasses both “target hopping” between different kinases and the discovery of unexpected kinase inhibitory activity in non-kinase drugs. In this review, we summarize recent advances in the repositioning of kinase inhibitors from medicinal chemistry and structural biology perspectives. We highlight the structural basis and binding modes that enable kinase polypharmacology and therapeutic exploitation of off-target activity. Furthermore, we discuss key structural optimization strategies used to convert initial repurposing hits into potent and selective anticancer agents. Overall, kinase-focused repurposing offers a rational, cost-efficient route to expand anticancer options by exploiting conserved kinase-recognition features and refining selectivity through structure-guided optimization. Translation remains constrained by tumor heterogeneity, incomplete target validation, computational-model bias, PK/PD and toxicity limitations, and regulatory and IP barriers. Integrating interpretable AI and multi-omics with kinome-wide profiling, direct target-engagement assays, patient-relevant models, and data sharing should improve patient stratification and accelerate clinical validation.

## 1. Introduction

Cancer remains one of the most pressing global health challenges and a leading cause of death worldwide. In 2024, nearly 21 million new cancer cases were reported worldwide, imposing a substantial economic burden on society. It is projected that more than 34.4 million new cancer cases will be diagnosed globally in 2050, with a 67% increase [1]. Despite significant advances in surgery, radiotherapy, chemotherapy, and immunotherapy, many cancer types still exhibit poor prognosis due to late diagnosis, tumor heterogeneity, acquired resistance, and dose-limiting toxicity of conventional treatments [2,3]. In addition, the translation of these novel drugs into clinical practice has been considerably slower than anticipated, with drug development typically requiring an average of approximately 13 years of research and development [4]. These limitations highlight the ongoing need for novel therapeutic strategies that are both effective and rapidly translatable to the clinic.

Protein kinases are important drug targets in oncology, serving as central regulators of fundamental cellular processes, including proliferation, survival, differentiation, and metabolism, and thus functioning as critical signaling hubs [5,6]. Aberrant kinase signaling is a hallmark of many cancers, driven by mutations, gene amplification, or dysregulated upstream signaling pathways. As a result, kinase inhibition has become one of the most successful approaches in targeted cancer therapy, leading to the development of numerous small-molecule kinase inhibitors over the past three decades [7]. However, challenges such as drug resistance, off-target toxicity, and low patient response rates continue to limit the long-term clinical efficacy of these agents [7,8]. The structural conservation of the kinase domain, particularly the ATP-binding pocket, provides a strong molecular basis for the cross-target activity of many drugs [9]. Nevertheless, subtle variations in pocket geometry, conformational dynamics, and local chemical environments determine inhibitor selectivity and binding affinity. These shared yet distinguishable features across the kinome create opportunities for identifying previously unrecognized therapeutic potential of known kinase inhibitors in different cancers [10].

Drug repurposing, also referred to as drug repositioning, reprofiling, or re-tasking, is an approach aimed at identifying new therapeutic indications for approved or investigational drugs beyond their originally intended medical uses [11]. It has emerged as a promising strategy for cancer treatment, with potentially lower cost and reduced development risk. Since the optimal dosing and the safety profile of existing drugs are already well established, this strategy entails reduced safety uncertainty and is typically associated with expedited regulatory approval and higher clinical success rates [12]. Recent work has expanded drug repurposing from serendipitous observations to systematic, evidence-driven pipelines. Contemporary oncology frameworks integrate target- and pathway-based reasoning, multi-omics and transcriptomic signatures, phenotypic and patient-derived organoid screening, large-scale pharmacological profiling, and structure-based or AI-enabled prediction to prioritize candidates and rational combinations [13,14].

Drug repurposing can be broadly categorized into four strategies. Classical drug repurposing refers to assigning a novel therapeutic indication to an approved, investigational, or discontinued drug without altering its chemical structure. Off-target-based drug repurposing involves the identification of an unanticipated molecular target that provides a mechanistic basis for the new therapeutic indication. When the original inhibitor molecule is retained while its target is switched to a different kinase within the kinase family, this strategy can be described as inter-kinase target hopping. Scaffold repurposing uses the chemical scaffold of a known drug or inhibitor as the starting point for subsequent medicinal chemistry optimization. Pharmacophore hybridization, in contrast, involves integrating structural elements from two or more parent molecules into a single molecular framework [11]. In addition, the evidence reported in the included studies was classified into four levels according to its strength: kinase inhibition assays, biochemical and cellular evidence, mechanistic in vivo efficacy, and clinical efficacy or regulatory approval for a new indication.

We searched PubMed, Web of Science and Google Scholar using combinations of the terms “drug repositioning”, “drug repurposing”, “kinase inhibitor”, “target hopping”, “structural optimization”, citing relevant studies published over the period from 2007 to 2026. In addition, eligible articles and relevant reviews were searched manually for additional primary studies. We included English-language primary studies that reported a defined compound, a newly proposed or experimentally supported kinase relationship, and an oncology-relevant phenotype or indication. Studies validated solely by computational simulation were excluded.

Recent advances in structural biology, machine learning, computational modeling, artificial intelligence (AI) and large-scale drug screening have significantly enhanced the ability to identify repositioning candidates [15]. Integrated approaches combining kinase profiling with systems pharmacology and network-based analyses further enable the prediction of drug–target interactions and pathway-level effects. Together, these developments have positioned drug repositioning as a powerful complement to traditional kinase inhibitor discovery.

In response to the urgent demand for novel anticancer therapies, drug repurposing has attracted growing attention in the field of oncology. In this review, we summarize current strategies and progress in drug repositioning for kinase inhibitor discovery in cancer therapy, focusing on structural mechanisms, computational and experimental approaches, and representative examples of repositioned kinase inhibitors. We also discuss the challenges and future perspectives for translating repositioned kinase-targeting drugs into clinically effective cancer therapies.

**Figure 1:**
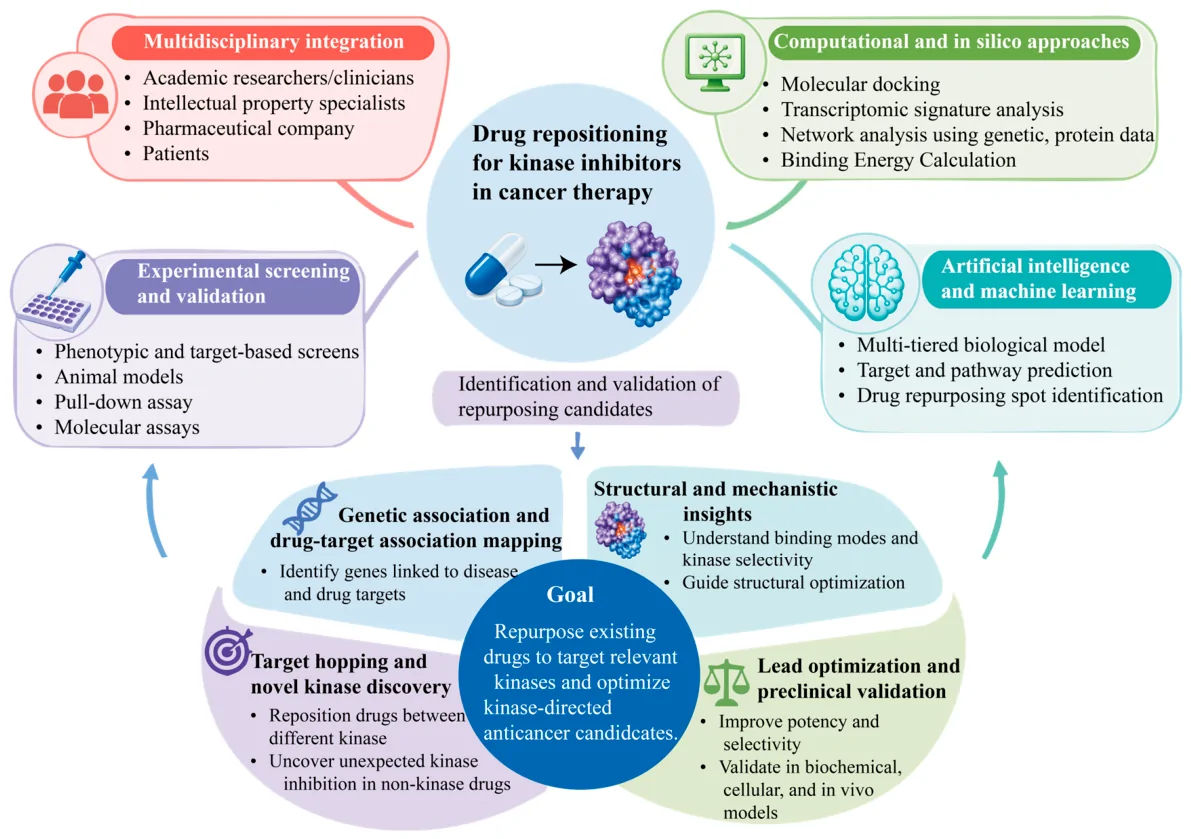
Integrated framework for kinase-focused drug repositioning in cancer research. Computational and in silico approaches, AI and machine learning, genetic and drug–target association mapping, and experimental screening collectively facilitate the identification of candidate drug–kinase relationships. Structural and mechanistic studies subsequently support target validation, elucidation of binding modes, target hopping, discovery of kinase activity in non-kinase drugs, and structure-guided lead optimization. This image was generated using Adobe Illustrator 2025 software.

As illustrated in Figure 1, kinase-focused drug repositioning is an iterative and multidisciplinary process that integrates computational and in silico prediction, AI and machine learning, genetic and drug–target association mapping, and experimental screening and validation. These complementary approaches facilitate the identification of previously unrecognized drug–kinase relationships through two principal routes: target hopping between different kinases and the discovery of kinase-directed activity in compounds originally developed for non-kinase targets. Subsequent biochemical, structural, and mechanistic studies establish direct target engagement, elucidate binding modes and selectivity determinants, and provide a rational basis for scaffold modification and lead optimization. Accordingly, this review first describes the structural features that enable kinase repositioning, then examines representative examples of kinase-to-kinase and non-kinase-to-kinase repositioning, and finally discusses computational and AI-driven discovery strategies together with the major challenges and future opportunities in this field.

## 2. The Structural Basis for Kinase Repositioning

### 2.1. Conservation of the Kinase Fold and the ATP-Binding Cleft

**Figure 2:**
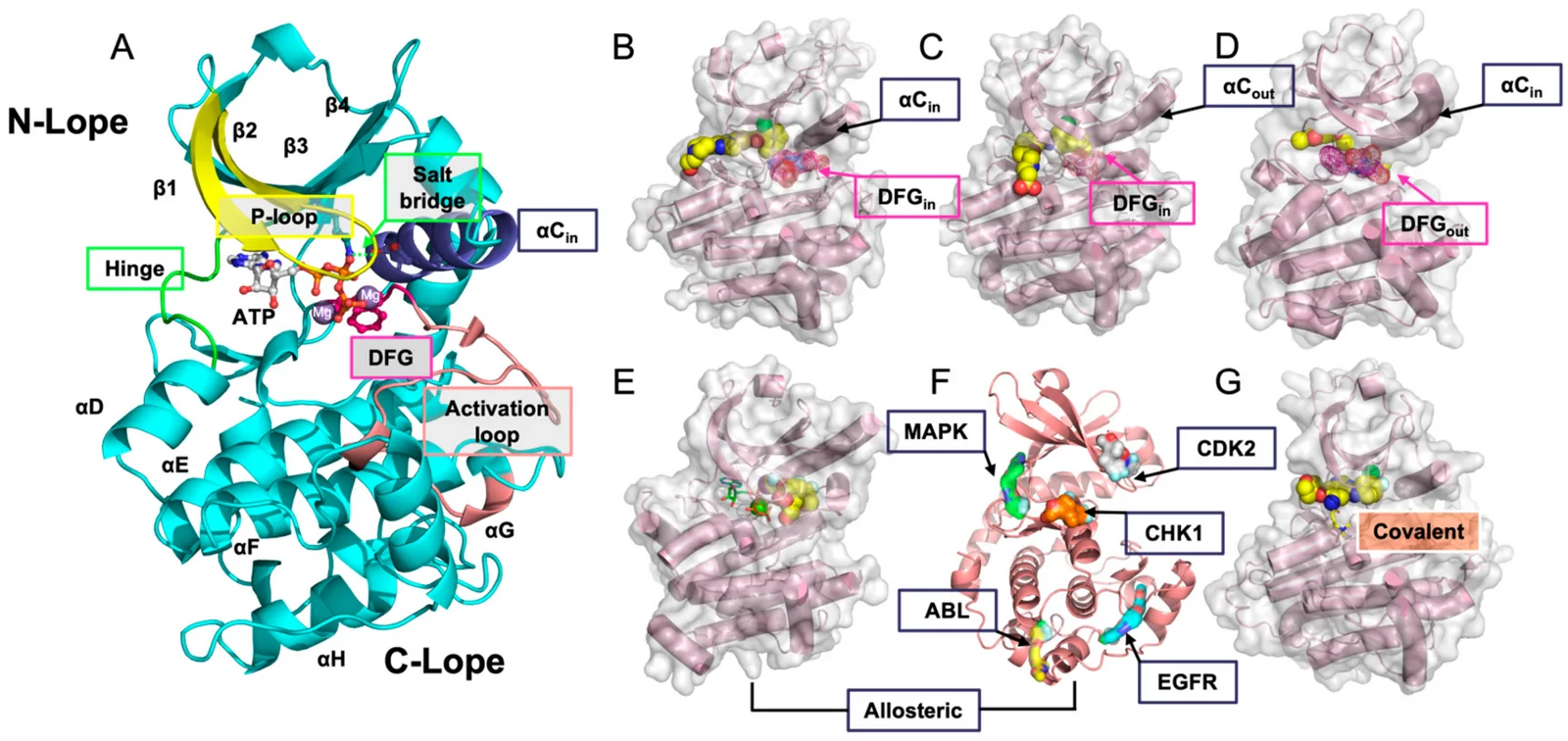
Structural features and binding modes of kinase inhibitors. (**A**), Overview of the kinase catalytic domain based on protein kinase A. (**B**–**G**), Representative type I (ABL, PDB: 2GQG), type I½ (EGFR–lapatinib, PDB: 1XKK), type II (BRAF–sorafenib, PDB: 5HI2), type III (MEK1–RDEA119–MgATP, PDB: 3E8N), type IV allosteric, and type V covalent (EGFR–afatinib, PDB: 4G5J) binding modes, respectively. This image was generated using PyMOL 3.0.3 software.

The structural basis for small-molecule kinase inhibitor repositioning lies in the highly conserved architecture of the kinase catalytic domain across the human kinome [9]. As exemplified by protein kinase A (PKA), the kinase fold adopts a characteristic bilobal architecture (Figure 2A) [16,17]. The smaller N-terminal lobe primarily comprises a five-stranded antiparallel β-sheet (β1–β5) and the conserved regulatory αC helix. In contrast, the larger C-terminal lobe is predominantly α-helical [17]. The cleft between these lobes forms a dynamic ATP-binding pocket, in which the adenine moiety of ATP interacts with backbone atoms in the hinge region [16,18]. Conservation of this pocket allows inhibitors developed for one kinase to retain affinity for other kinases with structurally compatible binding sites.

Key conserved elements include the glycine-rich P-loop (GXGXΦG), the AXK motif in the β3 strand, the αC helix, and the Asp-Phe-Gly (DFG) motif at the beginning of the activation loop. These elements collectively regulate ATP recognition, catalytic activity and kinase conformation. Their conservation underpins the cross-reactivity and multitargeting potential of many small-molecule inhibitors. Consequently, therapeutic scaffolds can be repositioned toward alternative kinases that share compatible structural and conformational features across different disease settings [19,20].

The ATP-binding cleft is not a uniform cavity but a set of contiguous interaction regions: the adenine-binding hinge, the ribose/solvent-exposed channel, the phosphate-binding region beneath the P-loop, and hydrophobic pockets around and behind the gatekeeper residue. Residues that contact the adenine and phosphate groups are strongly conserved, whereas gatekeeper-adjacent and solvent-front positions are more variable across the kinome. This conserved core allows a heterocyclic hinge-binding scaffold to transfer between kinases, while substituents directed toward the less-conserved subpockets can tune affinity and selectivity [21,22].

### 2.2. Structural Subregions Driving Selectivity and Repositioning Opportunities

Despite the overall conservation of the kinase fold, local geometry and stereoelectronic features within the ATP-binding pocket influence whether an inhibitor can be repurposed or optimized for another kinase [10]. Kinase drug repositioning therefore depends on both conserved anchoring interactions and target-specific structural variations.

For repositioning, compatibility should therefore be evaluated across the entire ATP pocket rather than from hinge similarity alone. The size and polarity of the gatekeeper residue control access to the hydrophobic back pocket, while differences in the P-loop, solvent-front residues, and the catalytic Lys-Glu/DFG network alter steric fit, hydration, and the preferred kinase conformation. An inhibitor may retain its hinge anchor in a new kinase yet gain or lose activity because one of these peripheral features changes; these same sites provide rational vectors for substituent growth or, when a suitably positioned nucleophilic residue is present, covalent targeting [21,22,23].

The hinge connects the N- and C-lobes and provides backbone carbonyl and amide groups that interact with ATP and ATP-competitive inhibitors. Ligands commonly form one to three hydrogen bonds with these backbone atoms, largely independent of side-chain variation. The conserved hinge therefore provides a universal anchoring site that supports cross-kinase activity and facilitates inhibitor repositioning without fundamental scaffold modification [24].

The glycine-rich P-loop covers the phosphate-binding region and helps position ATP or ATP-competitive inhibitors. The αC helix can adopt αC-in or αC-out conformations. In the αC-in state, the conserved β3-lysine–αC-glutamate salt bridge stabilizes the active kinase conformation. Disruption of this interaction promotes the αC-out state and remodels adjacent pockets, creating opportunities for selective or allosteric inhibitor binding [25].

The DFG motif switches between DFG-in and DFG-out conformations and thereby regulates the architecture of the activation loop. In the DFG-in state, the aspartate coordinates magnesium ions and supports ATP binding. In the DFG-out state, rearrangement of the phenylalanine exposes an adjacent hydrophobic pocket. Together with αC-helix movement, this conformational plasticity enables the type I, type I½, type II and allosteric binding modes shown in Figure 2B–G. Remote allosteric sites and covalent interactions further expand the mechanisms available for kinase inhibition and drug repositioning [25].

This pocket-centered view also explains why transferability differs among inhibitor classes. Type I ligands bind the ATP site in DFG-in conformations and can exploit the most conserved interactions, whereas type I½ and type II ligands additionally depend on inactive-state cavities generated by αC-helix and DFG rearrangements. Accordingly, a credible repositioning hypothesis should compare both residue-level pocket complementarity and the conformational ensemble accessible to the new kinase, because sequence identity or a single static structure alone may not reveal whether the required binding-competent state is populated [22,26].

## 3. Repositioning Between Different Kinases (Target Hopping)

A major structural basis for kinase-to-kinase repositioning is the evolutionary and structural conservation of the kinase catalytic domain. Many kinase inhibitors recognize conserved features of the ATP-binding cleft, particularly the hinge region and the conserved catalytic machinery surrounding the adenine-binding site [18,27]. Consequently, inhibitors developed against one kinase may retain measurable affinity for other kinases with compatible pocket geometry and conformational states [28]. However, cross-kinase activity is not determined by sequence similarity alone. Differences in gatekeeper residues, P-loop conformation, DFG orientation, solvent-exposed regions, and allosteric pockets can substantially alter binding affinity and selectivity [23,29]. Therefore, kinase-to-kinase repositioning reflects a balance between conserved recognition elements that permit binding and target-specific structural features that determine potency and selectivity [18,30].

**Figure 3:**
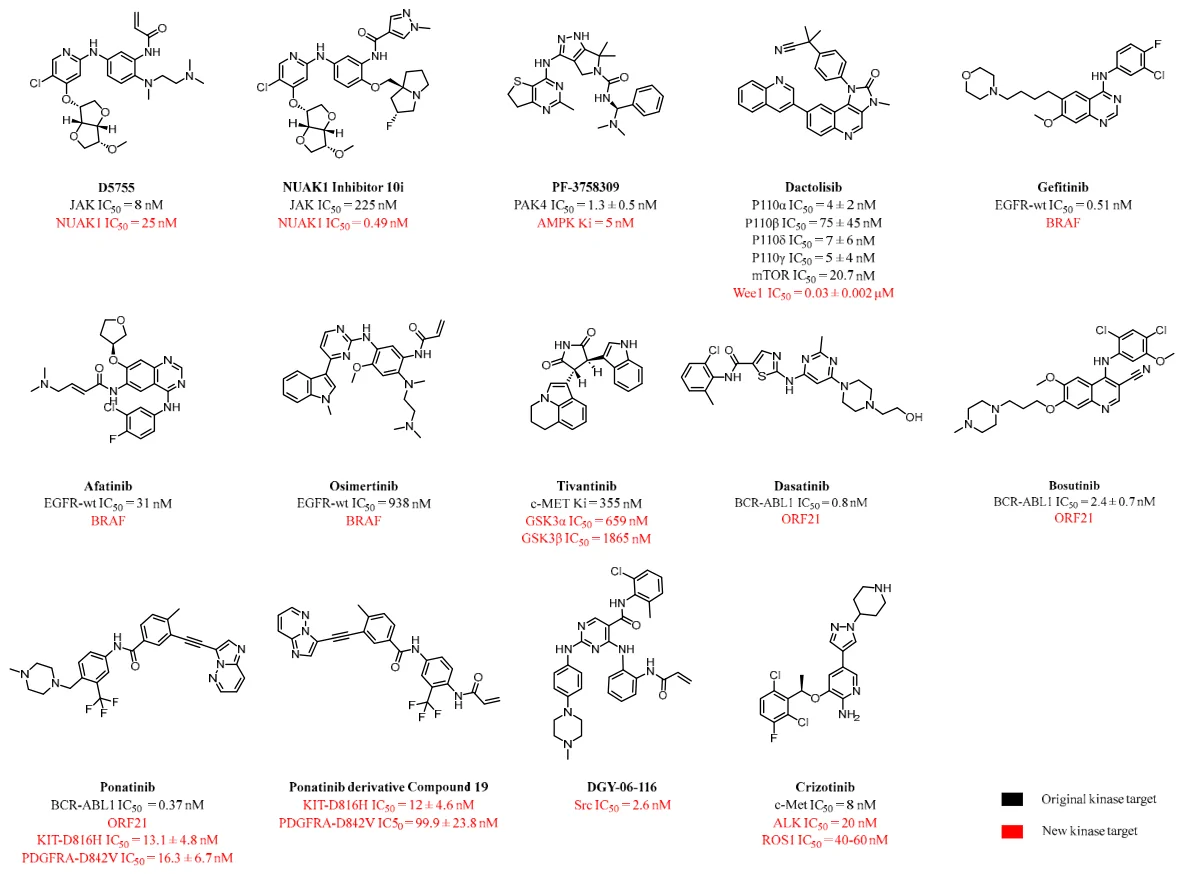
Representative examples of kinase-to-kinase repositioning through intrinsic cross-kinase activity or structure-guided scaffold optimization. The originally recognized kinase targets and their inhibitory activities are shown in black, whereas the newly identified or structurally redirected kinase targets are shown in red. This image was generated using Chemdraw22 software.

This section separates two routes that were previously grouped together: kinase-to-kinase repositioning uses the unchanged parent inhibitor against a new kinase-supported indication, whereas scaffold repurposing creates a new derivative and is categorized as medicinal-chemistry lead discovery. As summarized in Figure 3, several clinically developed inhibitors, including **dactolisib**, EGFR inhibitors, **tivantinib**, **dasatinib**, **bosutinib**, **ponatinib**, and **crizotinib**, exhibit potent activity against additional kinases beyond their originally recognized targets. In parallel, the conversion of **D5755** into the NUAK1 inhibitor **10i, ponatinib** into covalent KIT/PDGFRA inhibitors, and the **dasatinib**-derived scaffold into **DGY-06-116** illustrates how conserved kinase-binding motifs can be retained while target-specific interactions are introduced to redirect potency and improve selectivity. The comparison of inhibitory activities against the original and newly assigned kinase targets highlights both the structural transferability of ATP-site recognition and the importance of subsequent medicinal chemistry optimization. Accordingly, intrinsic polypharmacology of the unchanged molecule is discussed as target hopping, whereas rationally engineered derivatives are evaluated separately as new kinase-directed leads.

### 3.1. Scaffold Repurposing and Derivative Design

#### 3.1.1. NUAK1 Inhibitor

**Figure 4:**
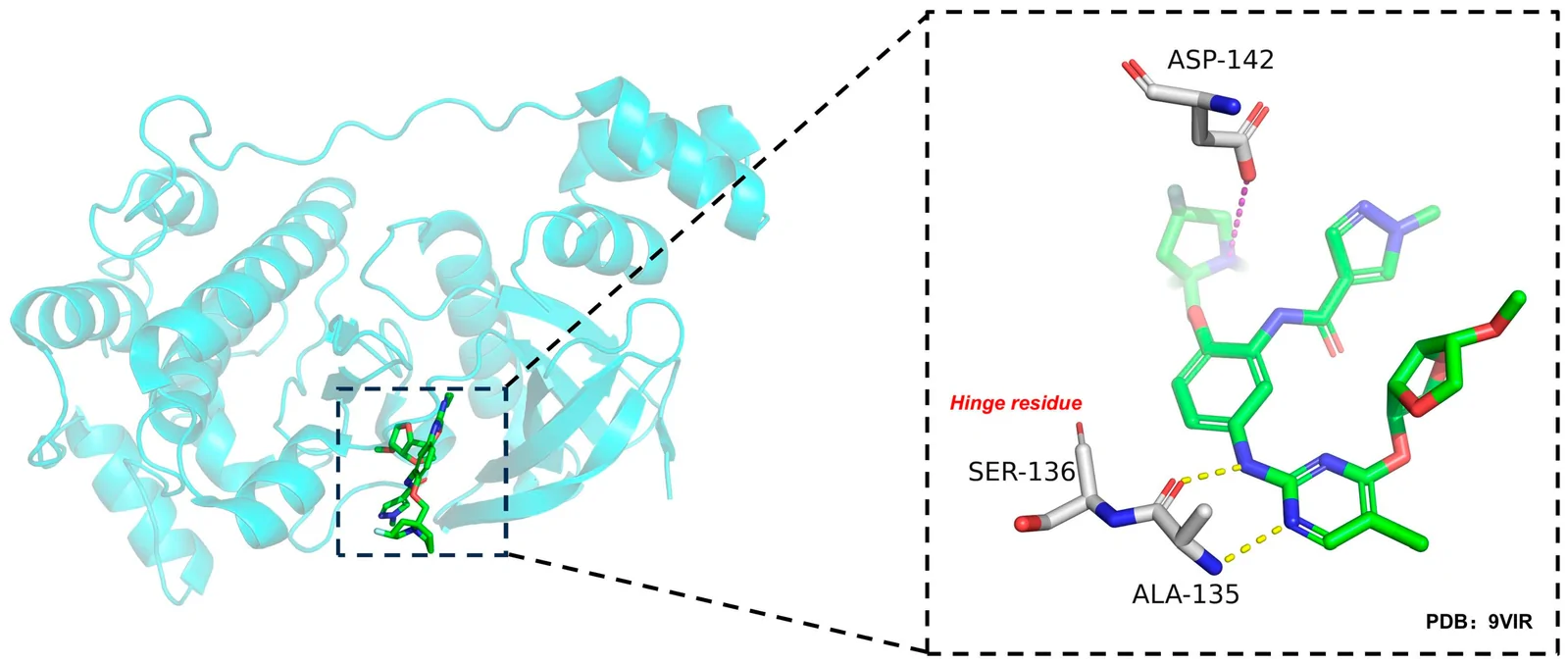
The co-crystal structure of **10i** bound to NUAK1-MARK3 kinase-domain chimera (PDB ID: 9VIR). Hydrogen bonds are shown as yellow dashed lines and salt bridge bond as magenta dashed lines. The image was generated using PyMOL 3.0.3 software.

**D5755** (Figure 3), a previously reported JAK3 inhibitor [31], was successfully redirected in selectivity from JAK3 to the oncogenic kinase NUAK1 through structural modification of its parent scaffold. Based on the structural similarities and differences between JAK3 and NUAK1, a novel derivative, compound **10i** (Figure 3), was rationally designed. Previous studies have found that the ATP-binding pockets of JAK3 and NUAK1 exhibit high structural similarity. However, their key pocket residues are different. **D5755** achieves covalent inhibition of JAK3 via modification of Cys909, while the equivalent residue at the corresponding pocket position of NUAK1 is the acidic residue Glu139 (Figure 4). Consequently, moieties with a preferential binding affinity for glutamate residues were incorporated to better accommodate the binding pocket of NUAK1. This compound exhibits potent inhibitory effects on TNBC cell growth in both *in vitro* and *in vivo* models [32].

#### 3.1.2. AMPK Inhibitor

**PF-3758309** (Figure 3), a compound originally designed as a PAK4 inhibitor, entered the terminated phase I study NCT00932126 [33]. Previous studies have demonstrated that **PF-3758309** exhibits therapeutic potential against pancreatic ductal adenocarcinoma, colorectal cancer and HIV infection. Although the Phase I clinical trial was terminated owing to unsatisfactory pharmacokinetic profiles observed, it serves as a valuable template for the discovery and development of targeted anticancer drugs. Therefore, Schneider et al. used a series of experiments to show that **PF-3758309** serves as a potent AMPK inhibitor, and its pharmacological activity is independent from the primary target PAK4. First, kinobeads assays demonstrated that **PF-3758309** has a higher binding affinity for AMPK subunits, particularly PRKAA1 than PAK4, and combined correlation tests and PAK4 knockout assays proved its PAK4-independent action. Second, molecular docking predicted a pose in which **PF-3758309** could occupy the ATP pocket of AMPKα, form hydrogen bonds with Glu96 and Val98, and make additional hydrophobic contacts. This modeled pose is hypothesis-generating and does not by itself demonstrate direct binding. **PF-3758309** as a single agent demonstrates robust therapeutic activity in lipogenic subtypes of PDAC; combinatorial treatment with ferroptosis inducers elicits striking synergistic anti-cancer responses, paving a new avenue for targeted combination therapy against pancreatic ductal adenocarcinoma [34].

Clinical translation is constrained by the parent compound’s development history: the phase I solid-tumor study NCT00932126 was terminated because of undesirable pharmacokinetic characteristics and the absence of a detectable dose–response relationship. Thus, renewed development for AMPK-directed pancreatic cancer would require a new exposure-target-engagement analysis rather than reliance on the earlier clinical-stage designation [35].

#### 3.1.3. KIT/PDGFRA Inhibitor

**Figure 5:**
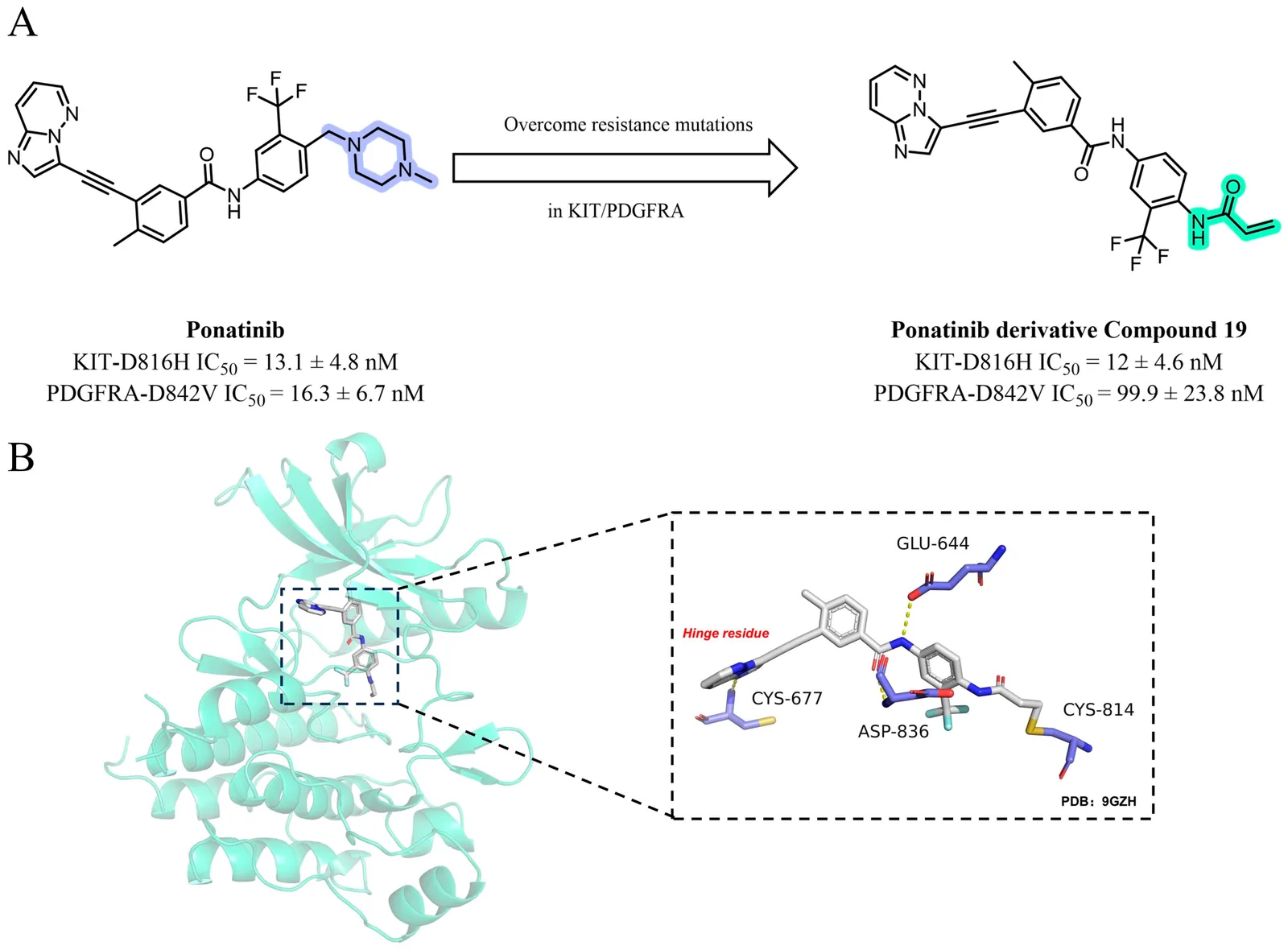
Selective covalent KIT/PDGFRA inhibitors compound **19**. (**A**) Design strategy for structural modification of compound **19**. (**B**) The co-crystal structure of **19** bound to PDGFRA-T674I (PDB ID: 9GZH). Hydrogen bonds were shown as yellow dashed lines. The image A was generated using Chemdraw22 software, image B was generated using PyMOL 3.0.3 software.

**Ponatinib** (Figure 3) represents a successful example of scaffold repurposing, where an originally developed BCR-ABL inhibitor was structurally repurposed toward mutant KIT and PDGFRA associated with gastrointestinal stromal tumors. By retaining the **Ponatinib** type II kinase-binding scaffold and introducing an electrophilic warhead targeting the poorly conserved cysteine residues (KIT-Cys788/PDGFRA-Cys814), Schulz et al. converted a promiscuous ATP-site inhibitor into selective covalent KIT/PDGFRA inhibitors compound **19** (Figure 3 and Figure 5A)**.** The imidazo[1,2-*b*]pyridazine core of **ponatinib** derivative occupies the hinge region and forms key hydrogen-bond interactions with the backbone of Cys673, while the hydrophobic moiety extends into the back pocket to establish stable hydrophobic interactions with Ile653, Leu783, and Ile808. Furthermore, the newly introduced acrylamide warhead is precisely oriented toward KIT-Cys788/PDGFRA-Cys814, enabling the formation of an irreversible covalent bond (Figure 5B). This unique dual-binding mode, characterized by “noncovalent ATP-pocket engagement combined with covalent cysteine anchoring”, successfully transforms the original BCR-ABL inhibitor into a selective covalent inhibitor targeting KIT/PDGFRA mutant kinases. These findings highlight the promising therapeutic potential of this derivative for the treatment of gastrointestinal stromal tumors (GIST), particularly those driven by drug-resistant KIT and PDGFRA mutations [36].

#### 3.1.4. Src Inhibitor

**Figure 6:**
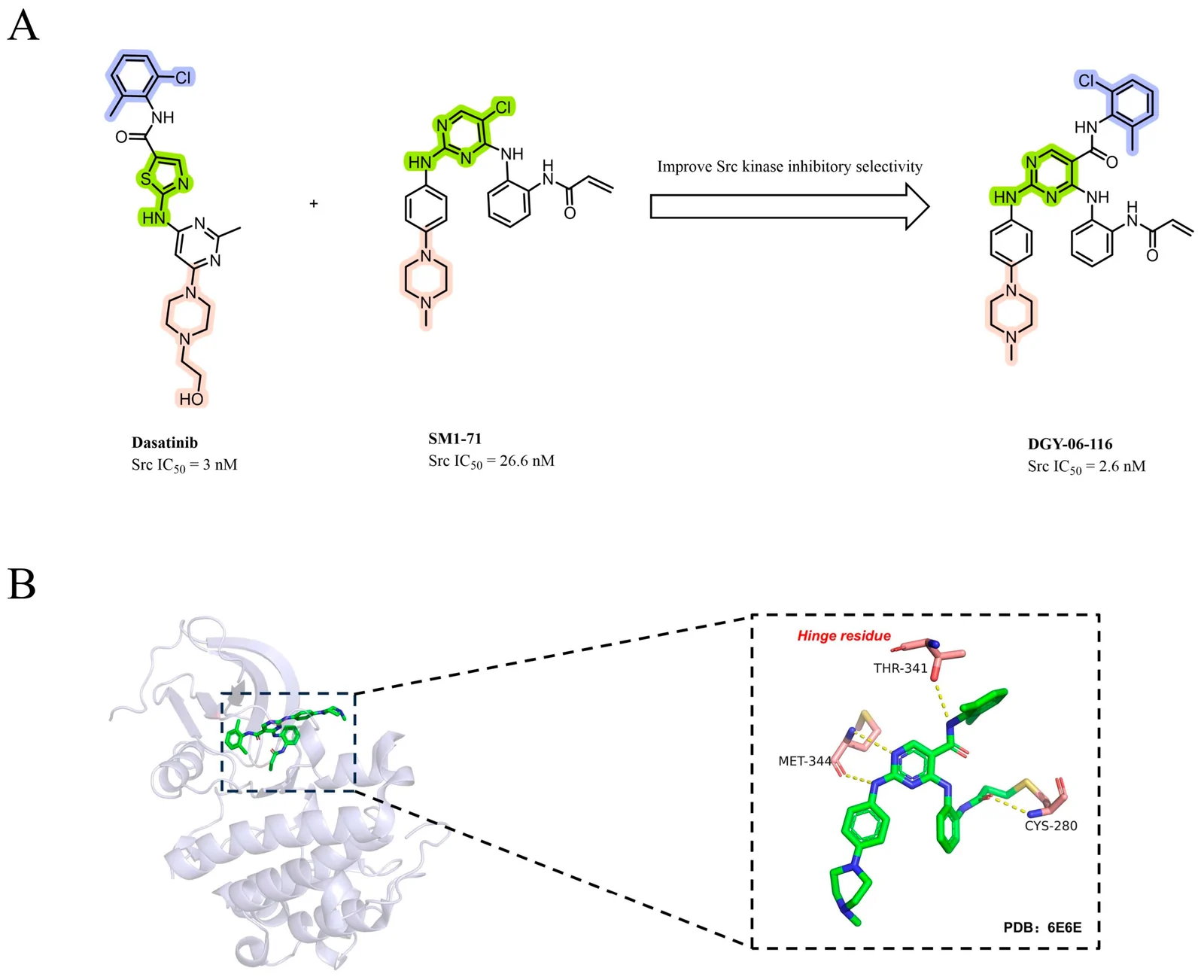
**DGY-06-116**, a **dasatinib**–**SM171** hybrid compound targeting Src kinase. (**A**) Structure-guided optimization strategy for the development of **DGY-06-116**. (**B**) The co-crystal structure of **DGY-06-116** bound to Src (PDB ID: 6E6E). Hydrogen bonds were shown as yellow dashed lines. The image A was generated using Chemdraw22 software, image B was generated using PyMOL 3.0.3 software.

**Dasatinib** (Figure 3), originally developed as a BCR-ABL family multi-kinase inhibitor for Philadelphia chromosome-positive leukemia [37]. Previous studies demonstrated that **dasatinib**, despite being originally developed as a BCR-ABL inhibitor, possessed intrinsic Src inhibitory activity mediated by conserved interactions with the ATP-binding pocket. Leveraging this latent kinase activity, researchers employed a structure-guided scaffold repurposing strategy to remodel the **dasatinib** framework, ultimately generating **DGY-06-116**, a selective covalent inhibitor targeting Src kinase. By hybridizing the **dasatinib** scaffold with the cysteine-reactive probe **SM1-71**, researchers introduced an acrylamide warhead capable of targeting the non-conserved Cys280 within the Src kinase P-loop. The **Src–DGY-06-116** co-crystal structure revealed that the inhibitor induces P-loop bending to enable covalent bond formation with Cys280, while forming hydrogen bonds with the hinge residue Met341, and hydrophobic interactions with Ile297 and Leu396 in the back pocket (Figure 6). This study demonstrates how structural modification of an existing kinase inhibitor scaffold can achieve target repositioning and enhanced kinase selectivity [38].

### 3.2. Existing Agents

#### 3.2.1. Wee1 Inhibitor

Pillai U et al. integrated machine learning–based in silico approaches with in vitro anticancer assays in triple-negative breast cancer (TNBC) models, employing multiple experimental strategies to identify **dactolisib** as a previously unrecognized inhibitor of Wee1 kinase. **Dactolisib** (Figure 3), a PI3K/mTOR kinase inhibitor with broad-spectrum antitumor activity, has advanced to Phase II clinical trials. Docking predicted an ATP-pocket pose for Wee1 with hydrogen bonds involving ASN 376, Asp386 and Cys379, along with thirteen non-hydrogen interactions involving VAL313, Val360, Phe433, Glu377, Ala326, Lys328, Ile374, Ile305 and Tyr378. In the reported functional assays, dactolisib showed Wee1-associated inhibition, which suggested that this ATP-binding pocket may serve as a critical site for repurposing existing drugs to target Wee1. **Dactolisib** not only avoided the dose-dependent toxicity associated with **adavosertib**, the first-generation Wee1 inhibitor but also exhibited superior efficacy against TNBC either as a single agent or in combination with **cisplatin**, and therefore has substantial potential for the development of novel anti-TNBC therapeutics [39].

#### 3.2.2. Mutant BRAF Inhibitor

Tyrosine kinase inhibitors (TKIs) targeting epidermal growth factor receptor (EGFR) are clinically applied to treat non-small cell lung cancer (NSCLC) harboring EGFR mutations. Nevertheless, Huo et al. employed PDX-based drug screening, genetic perturbation approaches, *in vitro* kinase assays, and molecular modeling studies demonstrated that these agents such as **afatinib**, **gefitinib** and **osimertinib** [40] (Figure 3) are also effective against NSCLC with the BRAF^G469V^ mutation. Molecular modeling analyses revealed that the ATP-binding pockets within the kinase domains of BRAF and EGFR share high structural homology. The G469V mutation induces a conformational rearrangement of the phosphate-binding loop (P-loop) in BRAF, which reduces the ATP-binding affinity of BRAF while enhancing the interaction between mutant BRAF and EGFR-TKIs, ultimately leading to TKI-mediated inhibition of mutant BRAF [41].

#### 3.2.3. GSK3α/β Inhibitor

**Tivantinib** (Figure 3) was originally developed as a highly selective non-ATP-competitive c-MET receptor tyrosine kinase inhibitor for targeted therapy of solid tumors, and has advanced into multiple clinical trials. Kuenzi et al. explored the drug repurposing potential of **tivantinib** based on its off-target effects, and showed that this agent potently inhibits glycogen synthase kinase 3α/β (GSK3α/GSK3β) with a moderate isoform preference for GSK3α, indicating its therapeutic potential against acute myeloid leukemia (AML). Studies have demonstrated that GSK3α is aberrantly overexpressed in AML and represents a promising novel therapeutic target for this malignancy. Compared with the pan-GSK3 inhibitor **lithium chloride** (**LiCl**), a traditional GSK3 inhibitor, **tivantinib** exhibits superior kinome selectivity and induces less undesired stabilization of β-catenin. Combination therapy assays revealed that **tivantinib** exerts robust synergistic antitumor activity with the BCL-2 inhibitor **ABT-199** (**venetoclax**). The combination treatment downregulates the expression of anti-apoptotic proteins MCL-1 and BCL-XL, and completely abolishes β-catenin accumulation induced by single-agent **tivantinib**. Therefore, monotherapy with tivantinib or its combination with ABT-199 supports preclinical evaluation in AML, but clinically achievable exposure, target engagement, safety, and efficacy for this new mechanism remain unestablished [42,43,44].

#### 3.2.4. ORF21 Inhibitor

The tyrosine kinase inhibitors **dasatinib**, **bosutinib** and **ponatinib** (Figure 3), originally indicated for chronic myeloid leukemia [45,46,47]. Previous studies demonstrated that the KSHV ORF21 protein exhibits noncanonical tyrosine kinase activity, suggesting a potential opportunity for repurposing clinically approved kinase inhibitors against KSHV-related diseases. Given the conserved architecture of the ATP-binding pocket across protein kinases, it was proposed that clinically approved TKIs could potentially be repurposed to target this viral kinase through cross-target inhibition. They directly bind to the ATP-binding pocket of KSHV ORF21 kinase to inhibit its autophosphorylation, and concurrently target cellular kinase pathways. They dually suppress KSHV lytic reactivation, progeny virion production and KSHV-driven tumor growth. These agents are potential candidates for drug repurposing against KSHV-associated diseases, including Kaposi’s sarcoma and lymphoma. **Dasatinib** is the most favorable candidate with potent anti-KSHV activity, low cytotoxicity and robust in vivo antitumor effects, whereas **ponatinib** and **bosutinib** are limited by severe toxicity and cell-type selectivity issues, respectively [48].

#### 3.2.5. ALK/ROS1 Inhibitor

**Figure 7:**
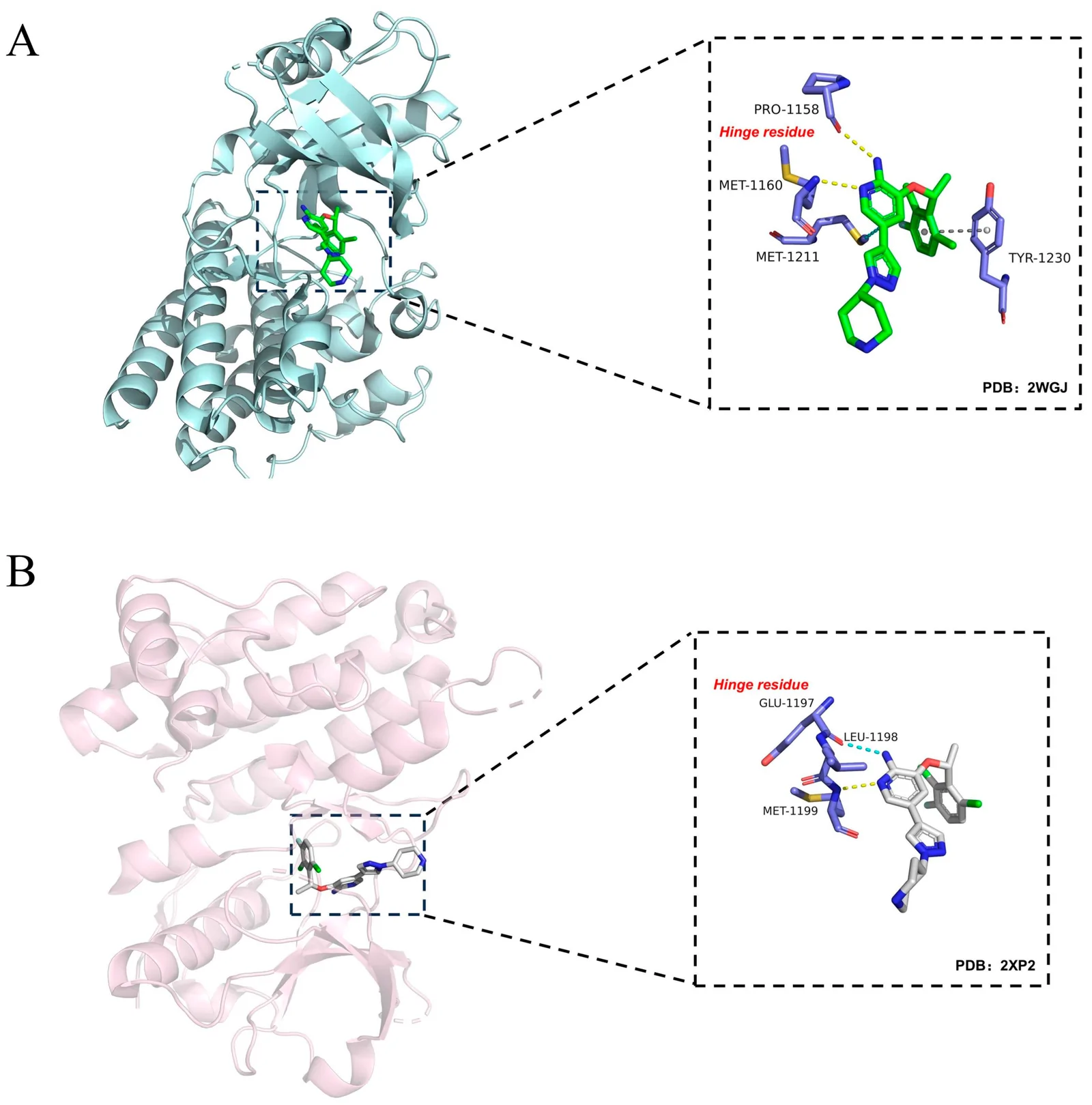
Structural basis of **crizotinib** targeting c-Met and ALK kinases. (**A**) The co-crystal structure of **crizotinib** bound to c-Met (PDB ID: 2WGJ). (**B**) The co-crystal structure of **crizotinib** bound to ALK (PDB ID: 2XP2). Hydrogen bonds were shown as yellow dashed lines, π–π stacking interaction as gray dashed lines and hydrophobic interactions as cyan dashed lines. The images of A, B were generated using PyMOL 3.0.3 software.

**Crizotinib** (Figure 3) is a successful example of kinase target repositioning, evolving from an originally developed MET inhibitor into a clinically validated ALK and ROS1 inhibitor [49,50,51]. It was originally designed for the treatment of solid tumors driven by c-MET amplification or mutation. During early kinase profiling studies, **crizotinib** was unexpectedly identified as a potent inhibitor of ALK [52]. Subsequent biochemical kinase assays and cellular studies confirmed direct ALK inhibition, particularly against EML4–ALK fusion-driven tumors [53]. The X-ray crystal structure of the ALK kinase domain in complex with **crizotinib** demonstrated that **crizotinib** directly occupies the ATP-binding pocket, forming hinge interactions and stabilizing the inactive kinase conformation. The binding of **crizotinib** to ALK is primarily mediated by hydrogen-bond interactions within the ATP-binding hinge region and hydrophobic stabilization provided by Leu1198. Compared with c-MET, ALK lacks the A-loop Tyr1230 residue that forms a π–π stacking interaction with the aromatic ring of **crizotinib**, resulting in a reduced binding stability (Figure 7). This structural difference may account for the weaker inhibitory potency of **crizotinib** against ALK compared with c-MET [50]. Owing to the structural homology between ALK and ROS1, **crizotinib** was further repositioned as a ROS1 inhibitor and approved for ROS1-rearranged NSCLC. Following drug repurposing, **crizotinib** has been approved for the treatment of multiple malignancies harboring kinase rearrangements, including ALK-rearranged inflammatory myofibroblastic tumor (IMT), ALK fusion-positive NSCLC, and ROS1-rearranged NSCLC. This case highlights how kinase profiling combined with structural biology can enable efficient repositioning of kinase inhibitors toward new oncogenic targets [51].

Taken together, these examples demonstrate that kinase-to-kinase repositioning proceeds through two complementary routes: direct exploitation of latent polypharmacology and structure-guided re-engineering of an established inhibitor scaffold. The conserved hinge-binding region and ATP-binding cleft provide a transferable interaction framework, allowing inhibitors developed for one kinase to retain measurable affinity for another. Meanwhile, variations in gatekeeper residues, accessible cysteines, P-loop conformations, and hydrophobic back pockets offer structural handles for redirecting potency and selectivity through scaffold modification or covalent warhead installation. Accordingly, successful target hopping requires more than the detection of cross-kinase binding; it depends on establishing target engagement, target-dependent cellular activity, and a therapeutically favorable selectivity profile in an appropriate molecular context. Kinome-wide profiling, structural biology, genetic perturbation, and disease-relevant models therefore constitute a convergent validation framework. Viewed from this perspective, target hopping is not merely the incidental reuse of a promiscuous kinase inhibitor, but a rational strategy for transforming conserved kinase recognition into clinically meaningful target selectivity and new therapeutic opportunities.

## 4. Repositioning from “Non-Kinase” to “Kinase” Targets

**Figure 8:**
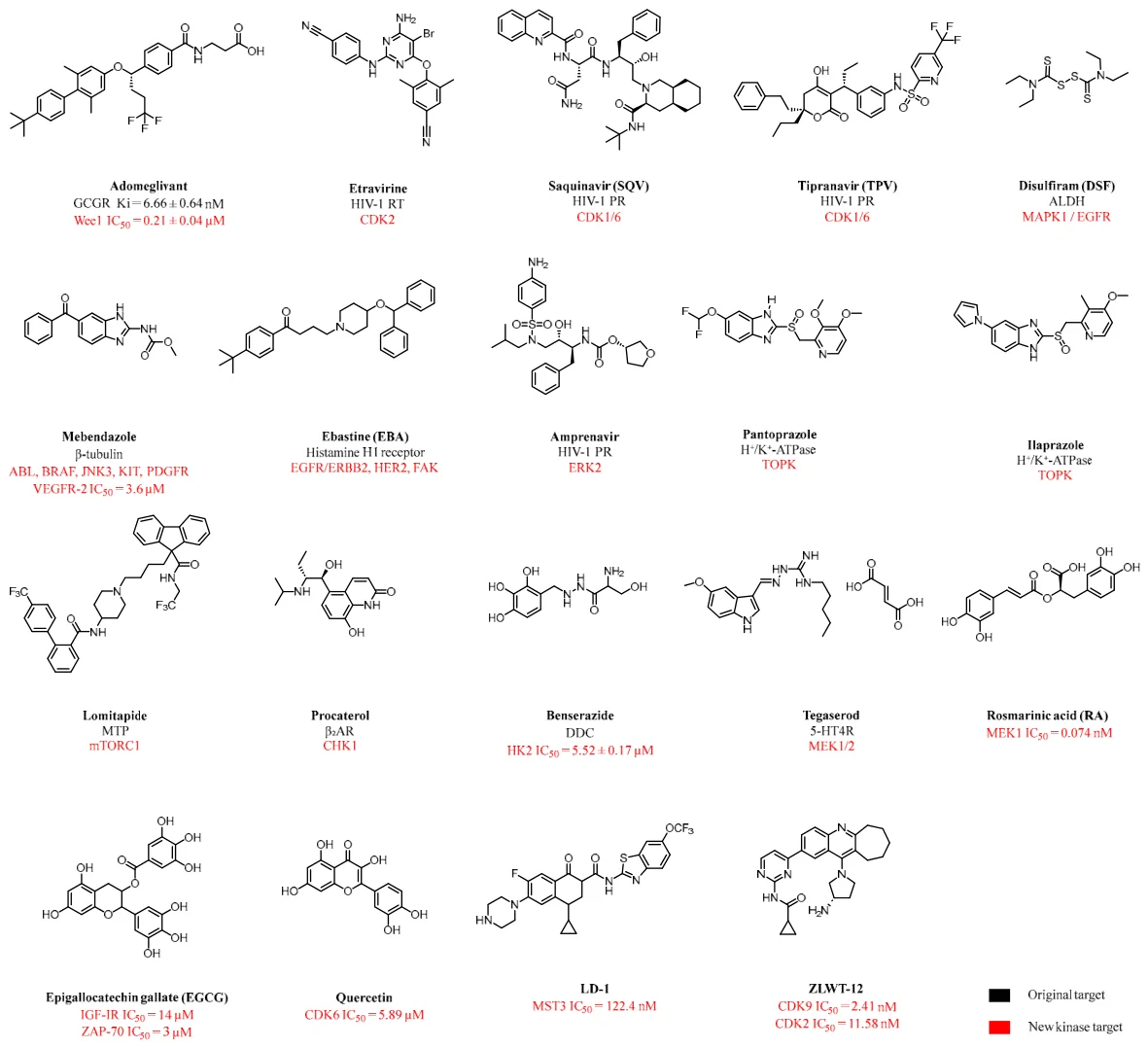
Representative examples of non-kinase-to-kinase repositioning through the identification of previously unrecognized kinase activity or structure-guided scaffold optimization. The original indications or pharmacological targets are shown in black, whereas the newly identified or structurally redirected kinase targets and their inhibitory activities are shown in red. This image was generated using Chemdraw22 software.

The identification of kinase activity in unchanged non-kinase drugs can support classical repositioning, whereas natural products without an established therapeutic indication and chemically modified derivatives are better regarded as target-discovery or lead-generation examples. As summarized in Figure 8, these distinct categories collectively expand the chemical space available for kinase inhibitor discovery. These examples encompass relatively focused kinase activities, such as the inhibition of Wee1 by **adomeglivant**, CDK2 by **etravirine**, ERK2 by **amprenavir**, and CHK1 by **procaterol**, as well as broader polypharmacological profiles represented by **mebendazole**, **ebastine**, **disulfiram**, **EGCG**, and **quercetin**. Beyond the direct repurposing of existing agents, non-kinase drugs can also provide starting scaffolds or pharmacophores for structure-guided kinase inhibitor development. For example, systematic modification of **tacrine**, a classical cholinesterase inhibitor, attenuated its original cholinesterase activity while generating **ZLMT-12**, a potent CDK2/9 dual inhibitor. Similarly, molecular hybridization of pharmacophoric elements derived from **riluzole** and **ciprofloxacin** led to the selective MST3 inhibitor **LD-1**. Collectively, these cases illustrate a continuum from the identification and validation of previously unrecognized kinase activity in existing compounds to the rational transformation of non-kinase pharmacophores into optimized kinase-directed leads, thereby providing both rapidly translatable drug candidates and unconventional scaffolds for anticancer drug discovery.

### 4.1. Existing Agents

**Adomeglivant** (Figure 8), with the glucagon receptor (GCGR) as its primary target, is a candidate drug for the treatment of type 2 diabetes. A recent study indicated that **adomeglivant** is capable of targeting Wee1 kinase to achieve anti-TNBC efficacy. **Adomeglivant** established two hydrogen bond interactions with Cys379 and Glu377, and eleven non-hydrogen interactions with Lys328, Phe433, Asp386, Tyr378, Ala326, and Val313. Its antitumor activity has been demonstrated in TNBC cell lines [39].

**Etravirine** (Figure 8), a non-nucleoside reverse transcriptase inhibitor initially developed for the treatment of HIV infection. Nair et al. employed multi-omics studies and molecular dynamics simulations found that **etravirine** directly interacts with the CDK2 kinase pocket. Mechanistically, **etravirine** inhibits CDK2, downregulates cyclin A2 expression, interrupts the cell cycle and triggers apoptosis in hepatocellular carcinoma cells, indicating its promising potential as an anti-hepatocellular carcinoma agent [54].

**Saquinavir** (SQV) and **tipranavir** (TPV) (Figure 8), originally developed as second-generation HIV protease inhibitors for HIV treatment, have subsequently been recognized for their broad-spectrum anticancer potential through diverse mechanisms. Using structural characterization and molecular docking analyses, Yu et al. identified cyclin-dependent kinases CDK1 and CDK6 as previously unrecognized targets of these antiviral agents, demonstrating their ability to directly bind and inhibit kinase activity. By suppressing CDK1/6 and synergistically activating the p53/p21/p16 senescence signaling pathway, they trigger G1-phase cell cycle arrest and ultimately produce therapeutic effects against cervical cancer [55].

**Disulfiram** (DSF) (Figure 8) is an FDA-approved drug for the treatment of alcohol dependence. Its classical mechanism of action involves the irreversible inhibition of hepatic aldehyde dehydrogenase (ALDH), which blocks the oxidative decomposition of acetaldehyde, the intermediate metabolite of alcohol metabolism. Consequently, **disulfiram** has been investigated for effects on pathways implicated in ALDH-positive NSCLC. Owing to its long-standing marketing history, extensive clinical application, well-established safety profile, and low cost, **disulfiram** has been investigated for anticancer repurposing. Liu et al. used computational modeling and cellular assays to propose that **disulfiram** acts on the EGFR binding site via key amino acid residues including Tyr727, Lys714, Glu746, and Thr725, and interacts with MAPK1 through residues such as Gln105, Lys54, Asp106, Ile53, Cys166, and Val39. Moreover, *in vitro* cellular experiments showed that **disulfiram** suppresses the proliferation of NSCLC cells [56].

Another study found that DSF directly binds to LCK via Cys20/23 and promotes its kinase activity, thereby enhancing T-cell effector responses and antitumor immunity of CD8^+^ T cells [57]. These findings demonstrated that **disulfiram** is not only an ALDH inhibitor but also a multi-target kinase inhibitor with favorable antitumor activity [56].

Nygren et al. screened 1600 clinically approved compounds and identified the anthelmintic drug **mebendazole** (MBZ) (Figure 8) as a potential anticancer agent for colorectal cancer. Subsequent *in vitro* binding studies revealed that MBZ directly targets multiple kinases, including ABL, BRAF, JNK3, KIT and PDGFR, while also exhibiting microtubule-disrupting activity. MBZ showed selective antitumor effects against colon cancer and demonstrated a favorable safety profile in clinical studies [58].

Computational drug repositioning further expanded the kinase-target landscape of MBZ. Dakshanamurthy et al. predicted VEGFR-2 as a potential target using a proteo-chemometric approach, which was subsequently confirmed by biochemical assays showing direct inhibition of VEGFR-2 kinase activity [59]. Further studies demonstrated that MBZ suppressed VEGF-induced VEGFR-2 phosphorylation at Y1175, reduced tumor angiogenesis, and inhibited tumor growth in medulloblastoma models [60]. Structural analysis revealed that MBZ occupies the VEGFR-2 kinase pocket through hydrogen bonding, hydrophobic interactions, and van der Waals forces, forming a stable inhibitory complex [61].

Collectively, MBZ represents a successful example of repositioning a non-kinase drug into a kinase inhibitor, supported by computational prediction, biochemical validation, structural analysis, and functional anticancer evaluation. These findings highlight the potential of MBZ as a VEGFR-2-targeting anticancer agent [58].

**Ebastine** (Figure 8), a second-generation H_1_-receptor antagonist clinically used for allergic rhinitis and urticaria, has recently been repositioned as a kinase-targeting anticancer agent. Yu et al. demonstrated through molecular docking and *in vitro/in vivo* studies that **ebastine** directly inhibits EGFR and ERBB2 by occupying their ATP-binding pockets, showing potent antitumor activity against EGFR/ERBB2-mutant lung adenocarcinoma. Moreover, **ebastine** enhances M1 macrophage polarization, providing an immunomodulatory advantage over conventional EGFR inhibitors [62]. Further studies revealed that **ebastine** may affect HER2- and FAK-associated signaling. In the HER2 study, molecular dynamics simulations predicted that ebastine could form hydrogen bonds within the ATP-binding site of the HER2 kinase domain and could form a possible π-cation interaction between Lys753 and one phenyl ring [63]. Seo et al. demonstrated that **ebastine** (EBA) directly targets and inhibits the kinase activity of focal adhesion kinase (FAK). EBA binds to FAK through hydrophobic interactions, hydrogen bonds and π-π stacking, thereby blocking the phosphorylation of FAK at Y397 and Y576/577 residues and suppressing the activation of downstream Src. Briefly, EBA exerts its antitumor activity by directly targeting the FAK kinase domain, leading to suppression of FAK autophosphorylation and subsequent inhibition of downstream Src signaling activation. In xenograft models, **ebastine** exhibited significant tumor growth inhibition with low systemic toxicity. Collectively, **ebastine** represents a notable example of repositioning an antihistamine into a multi-kinase inhibitor, with demonstrated activity against EGFR/ERBB2-driven lung cancer, HER2-resistant breast cancer, and FAK-dependent TNBC [64].

The study by Jiang et al. first demonstrated that **amprenavir** (Figure 8), an HIV-1 protease inhibitor, was evaluated as an ERK2 inhibitor. Molecular dynamics simulations predicted that **amprenavir** could occupy the ERK2 ATP-binding pocket through predicted hydrogen bonds with Lys41 and Arg54 and hydrophobic contacts with Ile18, Ala22, and Val26. The simulation supports a binding hypothesis rather than direct target engagement; separately, kinase assays showed that **amprenavir** directly inhibits the catalytic kinase activity of ERK2. Furthermore, *in vivo* experiments validated that **amprenavir** significantly suppresses the growth of breast cancer xenografts, indicating its therapeutic potential for anticancer treatment [65].

**Pantoprazole** (Figure 8), a proton pump inhibitor (PPI), has been repurposed as a non-kinase drug targeting T-cell-originated protein kinase (TOPK). Structure-based virtual screening identified **pantoprazole** as a binder of the ATP-binding pocket of TOPK. Docking analysis indicated that **pantoprazole** forms hydrogen bonds with Lys213 and Tyr264, and π–π stacking with Phe197, suggesting occupation of the catalytic ATP-binding cleft and potential competition with ATP. Microscale thermophoresis confirmed direct binding to TOPK with a *K*_d_ of 24.2 ± 2.32 μM. Functionally, **pantoprazole** inhibited TOPK-mediated phosphorylation of histone H3 (Ser10), indicating suppression of kinase activity. TOPK knockdown reduced **pantoprazole** sensitivity, supporting TOPK as its functional target, while **pantoprazole** suppressed colorectal cancer growth and tumor progression with low toxicity. Overall, **pantoprazole** represents a repurposed non-kinase drug that directly targets the ATP-binding pocket of TOPK and inhibits its oncogenic kinase activity [66]. The unbound systemic exposure at the standard dose is expected to be substantially below the *in vitro* Kd. This comparison does not exclude tumor or formulation-specific effects, but it identifies an exposure gap that must be addressed with dose-ranging, intratumoral PK, and pharmacodynamic target-engagement data before clinical translation.

**Ilaprazole** (Figure 8) is a PPI clinically indicated for the treatment of duodenal ulcer and reflux esophagitis. The study by Zheng et al. demonstrated that **ilaprazole** can directly target TOPK, a serine/threonine kinase that is aberrantly overexpressed in various tumors and governs tumor mitosis and proliferation, and has been recognized as a novel target for cancer therapy. Molecular docking analysis revealed that **ilaprazole** binds to TOPK through hydrogen bonds with Gly223 and Arg155, along with π–π stacking interactions involving Pro169 and Pro154, contributing to stable complex formation. Microscale thermophoresis (MST) confirmed the direct binding between **ilaprazole** and TOPK, while gene knockdown studies demonstrated TOPK dependency of its antitumor activity. *In vitro* and *in vivo* studies further validated the anticancer efficacy of **ilaprazole** with a favorable safety profile, supporting its potential as a TOPK-targeted therapeutic lead [67].

**Lomitapide** (Figure 8), a cholesterol-lowering agent, directly suppresses the kinase activity of mTOR and triggers autophagy, thereby inhibiting tumor cell proliferation and facilitating cancer cell death. It specifically binds to the ATP-binding catalytic core of mTORC1 by interacting with several residues while exhibiting no binding activity toward mTORC2. Thermal shift assays verified that **lomitapide** directly interacts with the recombinant mTOR kinase domain and reduces its thermal stability. *In vitro* kinase assays showed that **lomitapide** dose-dependently inhibits mTORC1-mediated S6K1 phosphorylation without affecting mTORC2-mediated Akt phosphorylation. In vivo studies further demonstrated potent antitumor activity with minimal toxicity, supporting its potential as an anticancer agent [68].

**Procaterol** (Figure 8) is a selective β_2_-adrenoceptor agonist, which is clinically applied for the prevention and treatment of asthma, asthmatic bronchitis, and wheezing caused by chronic obstructive pulmonary disease (COPD). Prema et al. employed in silico virtual screening and *in vitro* cellular assays; computational analyses suggested that **procaterol** could adopt a CHK1 active-site pose and favor CHK1 over CHK2. Molecular dynamics simulations supported the stability of the modeled complex. It is hypothesized that the compound may possess physicochemical and pharmacokinetic profiles amenable to drug development. Moreover, the co-treatment with **procaterol** and **cisplatin** significantly improves the antitumor activity against gastric adenocarcinoma through cell cycle blockade, as well as inhibition of cell proliferation and migration. It represents a promising combinatorial strategy for gastric cancer, which awaits further validation in animal models [69].

Originally used to treat Parkinson’s disease, **benserazide** (Figure 8), a dopa decarboxylase inhibitor, has been proven effective against colorectal cancer. Through hydrogen bond interactions, the pyrogallol moiety of **benserazide** targets the glucose-binding pocket of hexokinase 2 (HK2) and suppresses HK2 activity in a mixed competitive and non-competitive manner. With high binding affinity and good selectivity over HK1, HK4 and other isoenzymes, **benserazide** inhibits tumor glycolysis and triggers cell apoptosis, thereby producing therapeutic effects against colorectal cancer. Therefore, **benserazide** is a promising candidate for future anti-colorectal cancer therapy [70].

**Tegaserod** (Figure 8), a 5-hydroxytryptamine 4 receptor (5-HT4R) agonist, is used clinically for irritable bowel syndrome [71]. **Tegaserod maleate** has been reported to exert anti-proliferative effects on various malignancies; however, its activity and molecular mechanisms in gastric cancer remain elusive. Molecular docking predicted that tegaserod maleate could form contacts with MEK1 at residues Asp208, Met146 and Asp152, while establishing intermolecular interactions with MEK2 at the Gly81 and Gly83 positions [72]. *In vitro* kinase analysis demonstrated that **tegaserod maleate** potently inhibited MEK1/2 kinase activity. The antitumor efficacy of **tegaserod maleate** was confirmed in animal models. These findings suggest that this agent is a potential MEK1/2 inhibitor for the treatment or prevention of gastric cancer [72].

### 4.2. Natural Product-Derived Compounds

**Rosmarinic acid** (RA) (Figure 8), a natural polyphenolic compound, possesses inherent anti-inflammatory and antitumor properties. However, the precise molecular targets and mechanisms underlying its anticancer activity remain poorly understood. Kang et al. used multiple approaches, including microscale thermophoresis (MST) and CRISPR/Cas9 gene knockout assays to show that RA directly binds MEK1 with high affinity. This interaction suppresses the phosphorylation of MEK1 and its downstream ERK, thereby preventing excessive activation and functional dysfunction of T cells and ultimately potentiating antitumor immunity. RA displays superior MEK1 inhibitory potency compared with **trametinib**, a clinically approved MEK inhibitor. It could be developed as an adjuvant for existing immunotherapies or a novel agent to boost T cell-dependent antitumor immune responses [73].

**(-)-epigallocatechin 3-gallate** (**EGCG**) (Figure 8), the major polyphenol constituent of green tea, has attracted considerable interest for drug repurposing owing to its broad-spectrum anticancer and immunomodulatory activities [74]. Accumulating evidence validated that **EGCG** acts as a multitarget ATP-competitive kinase inhibitor, directly targeting RSK2, IGF-1R and ZAP-70.

Chen et al. identified RSK2 as a direct target of **EGCG** through virtual screening, which was subsequently confirmed by pull-down, ATP-competition, and molecular docking assays. **EGCG** bound to the ATP-binding sites of both RSK2 kinase domains and inhibited its activity in an ATP-competitive manner. RSK2 knockout studies further supported target dependency, highlighting the potential of **EGCG** for RSK2-targeted cancer chemoprevention [75]. Li et al. similarly demonstrated that **EGCG** directly inhibits IGF-1R kinase activity by occupying its ATP-binding pocket. **EGCG** specifically binds to the kinase domain of IGF-IR in its activated conformation, inserts into the ATP-binding pocket, and forms hydrogen bonds with key amino acid residues. By competing with ATP for the binding site, **EGCG** blocks the catalytic function of the receptor kinase. However, this study was limited to in vitro validation [76]. In addition, Shim et al. identified ZAP-70 as another direct target of **EGCG**, demonstrating ATP-competitive inhibition through binding to its kinase domain and supporting its potential application in leukemia treatment [77].

**Quercetin** (Figure 8), a widely distributed flavonoid in fruits and vegetables, possesses diverse biological activities, including anticancer and anti-inflammatory effects. Yousuf et al. identified **quercetin** as a potential CDK6 inhibitor through molecular dynamics simulations and binding free energy calculations. **Quercetin** was predicted to occupy the ATP-binding pocket of CDK6 and to form key hydrogen bonds with Asp102 and Asp163, which was further supported by in vitro kinase assays. Cellular studies showed that **quercetin** suppressed CDK6 expression, reduced cell viability and colony formation, and induced apoptosis in breast and lung cancer cells, highlighting its potential for CDK6-targeted cancer therapy [78].

### 4.3. Scaffold-Based Lead Discovery and Medicinal Chemistry Optimization

**Figure 9:**
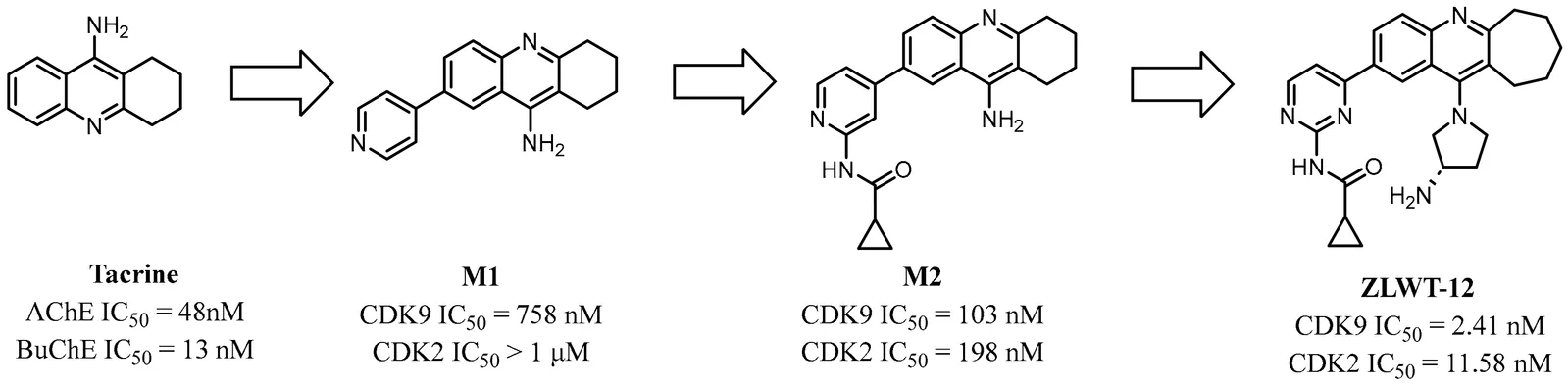
Design strategy for the **tacrine**-derived compound **ZLMT-12**. This image was generated using Chemdraw22 software.

**Tacrine** (1,2,3,4-tetrahydroacridin-9-amine), a classical cholinesterase (ChE) inhibitor, was the first FDA-approved therapeutic agent for Alzheimer’s disease (AD). Despite its clinical efficacy, **tacrine** was withdrawn from the market due to dose-dependent hepatotoxicity. In recent years, extensive efforts have been devoted to developing **tacrine**-based derivatives, many of which have shown promising anticancer properties. However, the retained ChE inhibitory activity of these derivatives may contribute to off-target effects and undesirable adverse events, representing a major limitation for their further clinical translation. Inspired by the potential of **tacrine** as a repurposable scaffold, our group performed scaffold-based structural modification and optimization, leading to the discovery of a series of highly potent CDK2/9 dual inhibitors. Our group performed systematic structural modifications targeting three key regions of the **tacrine** scaffold. First, the toxicity-associated free primary amine group was replaced with a chiral pyrrolidine amide side chain, which not only mitigated the potential hepatotoxicity associated with the parent scaffold but also established additional hydrogen-bonding interactions within the kinase binding site. Second, the saturated cycloalkyl ring of the tetrahydroacridine core was expanded to enhance cellular permeability and improve antitumor activity. Third, a pyrimidine heterocycle pharmacophore was introduced to construct a pharmacophore-hybridized molecule, thereby remodeling the interaction pattern between the compound and the ATP-binding pockets of CDK2 and CDK9 (Figure 9).

Through these structural optimizations, the lead derivative **ZLMT-12** (Figure 8) was identified. This compound exhibited a substantially reduced cholinesterase inhibitory activity compared with the parent **tacrine** scaffold while achieving nanomolar-range dual inhibition of CDK2 and CDK9. These findings demonstrate the potential of a scaffold-based drug repositioning strategy for kinase-directed lead discovery, transforming a neurodegenerative disease-related lead molecule into a potent anticancer CDK2/9 kinase inhibitor [79,80].

**Riluzole**, an inhibitor of glutamate release, was originally approved for the treatment of amyotrophic lateral sclerosis (ALS) [81]. **Ciprofloxacin**, a quinolone antibiotic that exerts its antibacterial activity by targeting bacterial topoisomerases and DNA gyrase, has been investigated for cancer therapy owing to the conserved catalytic mechanisms and structural similarities between bacterial and human topoisomerases [82]. Previously, we employed a drug repurposing–molecular hybridization strategy by integrating the pharmacophoric moieties of **riluzole**, an approved drug for ALS, and **ciprofloxacin**, a quinolone antibacterial agent, to design and synthesize a series of novel MST3 kinase inhibitors. Among these compounds, the lead compound **LD-1** was identified as a highly selective MST3 inhibitor. Structural studies revealed that **LD-1** occupied the ATP-binding pocket of MST3 kinase, in which the aminothiazole moiety derived from **riluzole** formed two key hydrogen bonds with the hinge region residue Leu102. Meanwhile, the quinolone carbonyl group from the **ciprofloxacin** scaffold established a hydrogen-bond interaction with Gly105, and the intramolecular amide carbonyl further interacted with surrounding water molecules within the binding pocket through hydrogen bonding. Functionally, **LD-1** exhibited potent antiproliferative activity *in vitro* by inducing apoptosis and G2/M cell-cycle arrest in hepatocellular carcinoma cells. *In vivo* studies of **LD-1** further demonstrated significant tumor growth inhibition with minimal observable toxicity. Our study provides a novel lead scaffold and a rational molecular design strategy for the development of targeted therapeutics against MST3-driven hepatocellular carcinoma [83].

Collectively, these studies show that non-kinase drugs and natural products constitute a chemically diverse and comparatively underexplored source of kinase-targeting scaffolds. Unlike kinase-to-kinase repositioning, this strategy often begins with an unexpected anticancer phenotype or a computationally predicted interaction, followed by identification of the responsible kinase target. Despite their distinct original pharmacology, many of these compounds converge on conserved kinase-recognition features, particularly the ATP-binding cleft, whereas others engage alternative catalytic pockets or regulatory residues, illustrating that kinase-directed activity can emerge from chemical architectures not originally optimized for the kinome. This diversity may offer new binding modes and polypharmacological profiles, but it also increases the risk of nonspecific activity, especially for broadly bioactive natural products. Consequently, docking or phenotypic activity alone is insufficient to establish repositioning: robust evidence should integrate direct-binding and biochemical assays with genetic target-dependency studies, pathway modulation, and *in vivo* efficacy. Furthermore, the concentrations required for kinase inhibition must be reconciled with clinically achievable exposure and the safety profile of the original indication. Thus, non-kinase-to-kinase repositioning is best regarded as a mechanism-driven lead-discovery strategy that converts established pharmacological agents into validated kinase-directed therapeutic candidates.

Overall, the examples fall into four non-equivalent categories. First, classical repositioning evaluates an unchanged approved or investigational drug in a new indication. Second, off-target discovery or kinase target hopping provides a mechanistic basis for repositioning when the same molecule is retained. Third, scaffold repurposing uses established scaffolds as starting points for medicinal chemistry optimization, including covalent-warhead installation. Fourth, pharmacophore hybridization yields new chemical entities by integrating structural elements from two or more parent molecules into a single molecular framework. Representative examples of this scaffold-based strategy and pharmacophore hybridization, including the development of **10i**, compound **19**, **DGY-06-116**, **ZLMT-12**, and **LD-1**, are summarized in Table 1. As summarized in Table 2, this direct-repositioning strategy encompasses both target hopping among kinases and the discovery of kinase-directed activity in drugs originally developed for non-kinase targets. Together, the two tables distinguish rational scaffold transformation from direct exploitation of intrinsic polypharmacology and provide a comparative overview of the original targets and indications, newly assigned kinase targets, proposed oncological applications, and current development status of the repositioning candidates.

## 5. Computational and AI-Driven Strategies in Kinase Repositioning

In recent years, the cost of new drug development has continued to increase; the expenditure required to bring a medicine from invention to market escalated to $2.8 billion in 2013, accompanied by a protracted research and development cycle of 10 to 15 years and a low success rate of only 2.01% for developing a new molecular entity [84]. Protein kinases represent one of the most prominent classes of oncogenic targets, yet traditional de novo inhibitor design is often bottlenecked by high attrition rates and extensive timelines.

AI-driven drug repositioning offers an effective framework that substantially reduces the time and financial burden of conventional drug discovery [85]. Computational and AI-driven strategies have emerged as powerful enablers for systematic drug repositioning, leveraging the exponential growth of biochemical and pharmacological datasets to extract actionable drug–kinase relationships at an unprecedented scale [86].

To bridge the gap between preclinical discovery and clinical translation, AI platforms leverage multi-tiered biological modeling to capture critical clinical hallmarks, such as tumor heterogeneity, dynamic microenvironment alterations, and metabolic diversity, which conventional *in vitro* assays often fail to recapitulate [87,88]. By integrating these complex physiological variables, AI allowed safety and efficacy assessments to be front-loaded, pre-emptively mapping tissue distribution, metabolite affinities, off-target liabilities, and optimal therapeutic windows to mitigate clinical attrition [85]. Ultimately, by cross-referencing these mechanistic insights with patient-specific multi-omic profiles, pharmacokinetics, and survival biomarkers, AI models can anticipate variations in patient responses. This enables the precise stratification of cohort populations most likely to benefit, while simultaneously generating high-throughput predictions for combination therapies, synthetic lethality, and strategies to circumvent drug resistance [89,90].

The paramount value of AI and computational modeling in drug repurposing lies in their capacity for data integration, connecting and integrating heterogeneous, multi-source big data to transform systems biology insights into actionable predictions of druggable targets or therapeutic inhibitors. Disease-driven big data integration uses multidimensional omics platforms, such as The Cancer Genome Atlas (TCGA) [91,92], to integrate genomics, transcriptomics, phenomics, proteomics, and metabolomics datasets. This holistic approach deciphers the underlying molecular mechanisms and hallmarks of complex malignancies, such as the constitutive activation of oncogenic signaling pathways [93].

To achieve systemic mechanism of action (MoA) predictions and identify novel target spaces, AI leverages multidimensional biological datasets through a rigorously defined, dual-pronged computational framework.

First, the framework deciphers direct molecular interactions through structure- and network-based drug-target prediction. By integrating the features of drug chemical scaffolds, 3D conformational similarities, and polypharmacological target interaction networks, machine learning models can scale up the screening of virtual compound libraries. Utilizing computational platforms such as ChemMapper, PREDICT, and SDTNBI [94,95,96], this approach effectively identifies previously undiscovered therapeutic targets and regulatory pathways at the structural level.

Second, the framework operates through phenotype-based signature reversal, aligning disease-specific transcriptomics with drug-induced perturbation profiles. This branch relies heavily on massive, genome-wide transcriptional datasets generated by large-scale profiling projects like CMap and LINCS [97,98]. By invoking bidirectional alignment and transcriptomic analysis tools including MANTRA, DSEA, and gene2drug [99,100,101,102], AI models can screen for FDA-approved small molecules predicted to reverse the oncogenic gene expression signatures back to a healthy state. As a representative validation of this phenotype-to-MoA strategy, the MANTRA platform—which employs gene set enrichment algorithms to cluster compounds based on shared pathway-regulatory effects—successfully identified anthelmintic drugs capable of suppressing the highly oncogenic PI3K/AKT/P70S6K signaling cascade [103].

By integrating multi-omics heterogeneous interaction networks through cutting-edge machine learning algorithms, such as CI-PMF [104] and multi-kernel Support Vector Machines [105,106] (multi-kernel SVM), macroscopic systems biology insights can be translated into highly credible, causally linked predictions of oncogenic druggable targets, thereby elucidating the precise relationships between disease structures and phenotypes.

These approaches can translate complex systems-biology and pharmacology data into credible drug-repositioning predictions and map disease-relevant structures, such as kinase pockets to clinical phenotypes and small-molecule activities.

Using AI classifiers and feature selection pipelines like Boruta allows for the precise screening of key topological and physicochemical features, including the hydrophobicity-related AlogP [107] and charge distribution-related Autocorrelation from an initial pool of 1444 high-dimensional molecular descriptors. This methodology mitigates the opacity associated with conventional molecular fingerprints, establishing an interpretable chemical-causal framework for drug repurposing predictions [108].

The implementation of AI to construct target-specific tailored classification matrices enables the automated screening of the top-performing classifier-feature pairings for 84 distinct human kinases. Within this framework, XGBoost [109] and RIDGE [110] emerge as the most robust models overall, facilitating high-accuracy prediction of ligand bioactivity profiles [111].

Machine learning has increasingly facilitated kinase drug repositioning by enabling systematic prediction and prioritization of previously uncharacterized drug–kinase interactions. De Simone et al. developed the Kinase drUgs mAchine Learning frAmework (KUALA), which combines kinase-specific ligand classification with binding-site similarity to generate a multi-target priority score (MTPS) and repurposing threshold for prioritizing reliable repositioning candidates. In an independent validation set of 50 drugs excluded from model training, KUALA correctly identified all experimentally validated kinase targets for 72% of the compounds, supporting its utility for kinase target profiling and repositioning [111]. Similarly, a kernel-based machine-learning framework predicted uncharacterized compound–kinase interactions, with experimental testing of 100 compound–kinase pairs showing a strong correlation between predicted and measured activities (r = 0.77) [112]. Structure-aware approaches such as iDTPnd further integrated molecular structural features and docking scores to identify previously unrecognized targets of approved kinase inhibitors, experimentally validating 10 novel interactions. Collectively, these studies demonstrate that AI-assisted ligand profiling, target prediction, and structure-based analysis can efficiently prioritize repurposable kinase ligands and uncover previously unrecognized target interactions, thereby providing a systematic strategy for kinase drug repositioning [113].

In addition to predicting structural docking, ligand similarity and binding affinity, systematic assessment of compounds’ physicochemical properties and ADME characteristics is an indispensable component of computational and AI-driven strategies for kinase repositioning. Physicochemical and absorption, distribution, metabolism, and excretion (ADME) profiling should be incorporated as an explicit filtering layer after computational prediction of a new drug-kinase interaction. Molecular weight (MW), lipophilicity (logP or pH-dependent logD), ionization, hydrogen-bond donor and acceptor counts, topological polar surface area (TPSA), and molecular flexibility influence aqueous solubility, membrane permeability, oral absorption, plasma protein binding, tissue distribution, metabolic turnover, and clearance. The Lipinski rule of five flags an increased risk of poor oral absorption or permeation when MW is greater than 500 Da, calculated logP is greater than 5, the number of hydrogen-bond donors is greater than 5, or the number of hydrogen-bond acceptors is greater than 10. Veber et al. further associated 10 or fewer rotatable bonds and a polar surface area of 140 Å^2^ or less with a higher probability of oral bioavailability [114,115]. These empirical thresholds support candidate prioritization rather than absolute exclusion, because active transport, formulation, route of administration, and high pharmacological potency can permit successful development outside conventional property space.

In kinase repositioning, these descriptors can be calculated alongside docking, ligand-similarity, and machine-learning target scores to identify candidates for which the proposed kinase potency is pharmacologically plausible at clinically tolerated exposure. SwissADME, pkCSM, and ADMETlab 2.0 provide complementary in silico predictions of physicochemical, pharmacokinetic, and toxicity endpoints, although predictions should be reconciled with measured human pharmacokinetic data from the original indication and experimentally validated for the proposed dose, formulation, and combination regimen [116,117,118]. A translational ranking workflow should therefore combine target-level evidence, including binding-site complementarity, predicted affinity, and network relevance, with exposure-level evidence, including unbound concentration relative to cellular potency, tissue distribution, and dose-limiting safety. Exposure at the site of action, target binding, and functional pharmacological activity are complementary requirements for testing a therapeutic mechanism [119]. This joint assessment is particularly important in drug repositioning because a dose established for the original indication may not sustain inhibition of the newly proposed kinase, and oncology combinations may alter exposure through Cytochrome P450 (CYP) or transporter-mediated drug–drug interactions.

## 6. Challenges and Future Perspectives

Cancer represents a major global public health and societal burden, accounting for approximately 16.8% of all-cause mortality and 22.8% of deaths attributable to noncommunicable diseases worldwide [120]. However, the development of anticancer drugs is characterized by prolonged timelines and substantial costs. Therefore, there is an urgent need to develop effective, safe, more affordable, and readily accessible anticancer agents. Drug repurposing offers a promising strategy to address the current challenges and limitations in anticancer drug development.

A major biological challenge is the structural and functional complexity of kinase signaling. Although kinase domains are evolutionarily conserved, subtle differences in ATP-binding pockets, allosteric sites, and activation-loop conformations can markedly affect inhibitor affinity and selectivity, limiting the direct transfer of kinase inhibitors across tumor types or disease contexts [10]. Moreover, kinase signaling operates within interconnected networks, in which inhibition of a single kinase can trigger compensatory signaling and feedback reprogramming, thereby reducing therapeutic durability and promoting resistance [121,122]. Tumor heterogeneity further complicates repurposing, as inter-patient genetic variation and intra-tumoral clonal evolution generate distinct kinase dependencies. Consequently, repurposed kinase inhibitors may benefit only molecularly defined patient subgroups, highlighting the importance of precision stratification [11].

Another limitation is the gap between computational prediction and experimental or clinical validation. Modern AI and machine learning-based approaches, including deep neural networks and transformer architectures, have significantly enhanced the prediction of drug–target interactions [123,124]. However, these models remain highly dependent on training dataset quality and are vulnerable to bias, overfitting, and limited generalizability when applied to underrepresented cancer types or rare mutational landscapes [125]. As a result, many computationally identified repurposing candidates fail during experimental validation, underscoring the need for robust multi-stage validation pipelines integrating in silico, *in vitro*, and *in vivo* models [11]. In addition, pharmacokinetic/pharmacodynamic (PK/PD) mismatches, off-target toxicity, and dose-limiting adverse effects may prevent clinically effective tumor exposure when drugs are transferred beyond their original indications [121]. These constraints are particularly critical in oncology, where achieving sufficient tumor exposure without systemic toxicity remains a major barrier.

Regulatory, intellectual property (IP), and economic barriers further restrict kinase inhibitor repurposing. Prior clinical data may streamline selected nonclinical or early clinical requirements, but repurposing does not automatically bypass phase I evaluation; the required program depends on the proposed dose, formulation, route, duration, combinations, cancer population, and whether existing exposure and safety data adequately bridge to the new use [126]. Moreover, limited regulatory incentives and fragmented approval pathways reduce commercial motivation. Patent expiration and overlapping IP claims further complicate the commercialization of repurposed kinase inhibitors, discouraging investment from pharmaceutical stakeholders [127].

Despite these challenges, several emerging technological and conceptual advances are expected to transform kinase inhibitor repurposing in cancer therapy. A major direction is the integration of systems pharmacology and network-based drug discovery. Network medicine approaches conceptualize diseases as perturbations in interconnected protein–protein interaction networks, enabling identification of shared pathway vulnerabilities across distinct diseases [121,122]. In parallel, integration of genomics, transcriptomics, proteomics, and phosphoproteomics can further identify context-specific kinase dependencies and improve the prioritization of repurposing candidates [123,125]. AI and machine learning, particularly graph neural networks and transformer-based models, offer powerful tools for integrating heterogeneous biological data to predict drug–target interactions at scale. However, future progress will depend on improving interpretability, addressing dataset imbalance, and incorporating iterative experimental feedback loops to refine predictive accuracy [125]. More physiologically relevant experimental systems, including iPSCs, organoids, and 3D tumor models, may further improve the validation of repurposed kinase inhibitors by better recapitulating tumor heterogeneity and the tumor microenvironment [128,129].

Finally, collaborative frameworks integrating academia, industry, regulatory agencies, and patient organizations are essential. Successful repurposing requires access to compound libraries, shared datasets, and translational expertise distributed across stakeholders. Precompetitive collaboration models and data-sharing initiatives have been shown to significantly enhance the efficiency of repurposing pipelines and reduce translational bottlenecks [130]. Strengthening this workflow will be critical for accelerating kinase inhibitor repurposing from computational prediction to clinical application.

## 7. Conclusions

In conclusion, the collected evidence supports kinase-focused drug repurposing as a productive but still predominantly preclinical strategy for cancer drug discovery. Across the 27 representative cases summarized in Table 1 and Table 2, five are structure-guided derivatives or pharmacophore-hybridized leads and 22 are agents directly reassigned through newly recognized kinase activity. These examples span four complementary modes: classical repurposing, off-target-based repurposing including inter-kinase target hopping, scaffold repurposing, and pharmacophore hybridization, and involve targets ranging from Wee1, AMPK, and CDKs to Src, ALK/ROS1, TOPK, and MEK1/2. Evidence strength extends from computational or cellular observations to biochemical, structural, mechanistic *in vivo*, and clinical validation. Notably, 26 of the 27 cases remain preclinical, whereas crizotinib provides the clearest clinical proof of concept through its approved ALK- and ROS1-directed indications. Collectively, drug repurposing offers a powerful and cost-efficient strategy for kinase inhibitor discovery in cancer therapy. These findings indicate that conserved kinase-binding features can enable efficient target transfer, but clinical success depends on direct target engagement, disease-context dependency, achievable exposure, selectivity, and safety. Integrating interpretable AI, multi-omics, kinome-wide profiling, and patient-relevant models should improve candidate prioritization and accelerate rigorous clinical validation, thereby enabling a more precise and rational framework for kinase-targeted drug repurposing and ultimately accelerating the development of effective cancer therapies.

## Figures and Tables

**Table 1:** Representative kinase-targeting leads developed through structure-guided modification or pharmacophore hybridization of existing drugs and pharmacologically characterized scaffolds.

Parent Drug	Derivatized Drug	Original Target	New Kinase Target	Original Indication	New Indication	Development Status	Evidence Level	Reference
**D5755**	**10i**	JAK3	NUAK1	hematological malignancies	TNBC	Preclinical	mechanistic *in vivo* efficacy	[31,32]
**Ponatinib**	**19**	BCR-ABL	KIT/PDGFRA	CML, ALL	GIST	Preclinical	biochemical and cellular evidence	[36]
**Dasatinib**	**DGY-06-116**	BCR-ABL	Src	Philadelphia chromosome-positive leukemia	Various cancer	Preclinical	biochemical and cellular evidence	[38]
**Tacrine**	**ZLMT-12**	—	CDK2/9	AD	CRC	Preclinical	mechanistic *in vivo* efficacy	[79,80]
**Riluzole**/**Ciprofloxacin**	**LD-1**	—	MST3	ALS/antibacterial	Hepatocellular carcinoma	Preclinical	mechanistic *in vivo* efficacy	[81,82,83]

**Table 2:** Representative drugs directly repositioned through the identification of previously unrecognized kinase targets.

Drug	Original Pharmacological Target	New Kinase Target	Original Indication	New Indication	Development Status	Evidence Level	Reference
**PF-3758309**	PAK4	AMPK	Pancreatic ductal adenocarcinoma, colorectal cancer, HIV infection	Pancreatic ductal adenocarcinoma	Parent clinical program terminated	mechanistic *in vivo* evidence	[33,34]
**Dactolisib**	PI3K/mTOR	Wee1	Bladder cancer, pancreatic cancer	TNBC	Preclinical	biochemical and cellular evidence	[39]
**Tivantinib**	c-MET	GSK3α/GSK3β	Various solid tumors	AML	Preclinical	mechanistic *in vivo* efficacy	[42,43,44]
**Dasatinib**	BCR-ABL	ORF21	Philadelphia chromosome-positive leukemia	KSHV-associated diseases, including Kaposi’s sarcoma and lymphoma	Preclinical	mechanistic *in vivo* efficacy	[48]
**Crizotinib**	c-MET	ALK/ROS1	solid tumors driven by c-MET amplification or mutation	ALK fusion-positive NSCLC, advanced ROS1-rearranged NSCLC	Approved	clinical efficacy and approval for ALK/ROS1 indications	[49,50,51,52,53]
**Adomeglivant**	GCGR	Wee1	type 2 diabetes	TNBC	Preclinical	biochemical and cellular evidence	[39]
**Etravirine**	HIV-1 RT	CDK2	HIV infection	Hepatocellular carcinoma	Preclinical	biochemical and cellular evidence	[54]
**Saquinavir**	HIV-1 PR	CDK1/6	HIV infection	Cervical cancer	Preclinical	mechanistic *in vivo* evidence	[55]
**Tipranavir**	HIV-1 PR	CDK1/6	HIV infection	Cervical cancer	Preclinical	mechanistic *in vivo* evidence	[55]
**Disulfiram**	ALDH	MAPK1/LCK	Alcohol dependence	Various cancer	Preclinical	biochemical and cellular evidence	[56,57]
**Mebendazole**	β-tubulin	ABL/BRAF/JNK3/KIT/PDGFR/ VEGFR-2	Anthelmintic	CRC	Preclinical	kinase inhibition assay; biochemical and cellular evidence	[58,59,60,61]
**Ebastine**	Histamine H1 receptor	EGFR/ERBB2/HER2/FAK	Antiallergic	Lung adenocarcinoma, breast cancer	Preclinical	mechanistic *in vivo* evidence	[62,63,64]
**Amprenavir**	HIV-1 PR	ERK2	HIV infection	Breast cancer	Preclinical	mechanistic *in vivo* evidence	[65]
**Pantoprazole**	H^+^/K^+^-ATPase	TOPK	Active gastric and duodenal ulcers, moderate to severe reflux esophagitis	CRC	Preclinical	mechanistic *in vivo* evidence	[66]
**Ilaprazole**	H^+^/K^+^-ATPase	TOPK	Active gastric and duodenal ulcers, reflux esophagitis	CRC	Preclinical	mechanistic *in vivo* evidence	[67]
**Lomitapide**	MTP	mTORC1	Antihyperlipidemic	CRC	Preclinical	mechanistic *in vivo* evidence	[68]
**Procaterol**	β_2_AR	CHK1	COPD	Gastric cancer	Preclinical	biochemical and cellular evidence	[69]
**Benserazide**	DDC	HK2	Parkinson’s disease	CRC	Preclinical	mechanistic *in vivo* evidence	[70]
**Tegaserod**	5-HT4R	MEK1/2	Irritable bowel syndrome	Gastric cancer	Preclinical	mechanistic *in vivo* evidence	[71,72]
**Rosmarinic acid**	—	MEK1	anti-inflammatory and antitumor	Various cancer	Preclinical	mechanistic *in vivo* evidence	[73]
**EGCG**	—	RSK2/IGF-1R/ZAP-70	Broad-spectrum antitumor activity, immunomodulatory effects	Breast cancer, cervical cancer, leukemia	Preclinical	biochemical and cellular evidence	[74,75,76,77]
**Quercetin**	—	CDK6	anticancer, anti-inflammatory	Various cancer	Preclinical	biochemical and cellular evidence	[78]

## Data Availability

No new data were created or analyzed in this study. Data sharing is not applicable to this article.

## Abbreviations

Abbreviations: ABL, Abelson tyrosine-protein kinase; AD, Alzheimer’s disease; ADME, absorption, distribution, metabolism, and excretion; AI, artificial intelligence; ALDH, aldehyde dehydrogenase; ALK, anaplastic lymphoma kinase; ALL, acute lymphoblastic leukemia; ALS, amyotrophic lateral sclerosis; AML, acute myeloid leukemia; AMPK, AMP-activated protein kinase; ATP, adenosine triphosphate; β_2_AR, β_2_-adrenergic receptor; BCL-2, B-cell lymphoma 2; BCL-XL, B-cell lymphoma-extra large; BCR-ABL, BCR::ABL1 fusion protein; BRAF, B-Raf proto-oncogene serine/threonine kinase; CDK, cyclin-dependent kinase; ChE, cholinesterase; CHK1/2, checkpoint kinases 1 and 2; CI-PMF, causal inference–probabilistic matrix factorization; CMap, Connectivity Map; CML, chronic myeloid leukemia; COPD, chronic obstructive pulmonary disease; CRC, colorectal cancer; CRISPR, clustered regularly interspaced short palindromic repeats; CYP, cytochrome P450; DDC, DOPA decarboxylase; DFG, Asp-Phe-Gly; DNA, deoxyribonucleic acid; DSEA, drug-set enrichment analysis; DSF, disulfiram; EBA, ebastine; EGCG, (−)-epigallocatechin-3-gallate; EGFR, epidermal growth factor receptor; EML4, echinoderm microtubule-associated protein-like 4; ERBB2, Erb-B2 receptor tyrosine kinase 2; ERK/ERK2, extracellular signal-regulated kinase/extracellular signal-regulated kinase 2; FAK, focal adhesion kinase; FDA, Food and Drug Administration; GCGR, glucagon receptor; GIST, gastrointestinal stromal tumor; GSK3, glycogen synthase kinase 3; GXGXΦG, glycine-rich P-loop motif; HER2, human epidermal growth factor receptor 2; HIV, human immunodeficiency virus; HIV-1 PR, HIV-1 protease; HIV-1 RT, HIV-1 reverse transcriptase; HK, hexokinase; IGF-1R/IGF-IR, insulin-like growth factor 1 receptor; IMT, inflammatory myofibroblastic tumor; IP, intellectual property; iPSC, induced pluripotent stem cell; JAK3, Janus kinase 3; JNK3, c-Jun N-terminal kinase 3; KIT, KIT proto-oncogene receptor tyrosine kinase; KSHV, Kaposi’s sarcoma-associated herpesvirus; KUALA, Kinase drUgs mAchine Learning frAmework; LCK, lymphocyte-specific protein tyrosine kinase; LINCS, Library of Integrated Network-Based Cellular Signatures; MANTRA, Mode of Action by Network Analysis; MAPK1, mitogen-activated protein kinase 1; MBZ, mebendazole; MCL-1, myeloid cell leukemia 1; MEK, mitogen-activated protein kinase kinase; MET/c-Met, MET proto-oncogene receptor tyrosine kinase; MoA, mechanism of action; MST, microscale thermophoresis; MST3, mammalian sterile 20-like kinase 3; MTP, microsomal triglyceride transfer protein; MTPS, multi-target priority score; MW, molecular weight; NSCLC, non-small-cell lung cancer; NUAK1, NUAK family SNF1-like kinase 1; ORF21, open reading frame 21; PAK4, p21 (RAC1)-activated kinase 4; PDAC, pancreatic ductal adenocarcinoma; PDB, Protein Data Bank; PDGFR, platelet-derived growth factor receptor; PDGFRA, platelet-derived growth factor receptor alpha; PDX, patient-derived xenograft; PI3K, phosphoinositide 3-kinase; PK/PD, pharmacokinetics/pharmacodynamics; PKA, protein kinase A; PPI, proton pump inhibitor; PRKAA1, protein kinase AMP-activated catalytic subunit alpha 1; RA, rosmarinic acid; ROS1, ROS proto-oncogene 1 receptor tyrosine kinase; RSK2, ribosomal protein S6 kinase 2; S6K1, ribosomal protein S6 kinase beta-1; SDTNBI, systematic drug-target network-based inference; SNF1, sucrose non-fermenting 1; SQV, saquinavir; SVM, support vector machine; TCGA, The Cancer Genome Atlas; TCR, T-cell receptor; TKI, tyrosine kinase inhibitor; TNBC, triple-negative breast cancer; TOPK, T-LAK cell-originated protein kinase; TPSA, topological polar surface area; TPV, tipranavir; VEGF, vascular endothelial growth factor; VEGFR-2, vascular endothelial growth factor receptor 2; XGBoost, extreme gradient boosting; ZAP-70, zeta-chain-associated protein kinase 70.
